# Pre-fusion AAA+ remodeling of target-SNARE protein complexes enables synaptic transmission

**DOI:** 10.1101/2024.10.11.617886

**Published:** 2024-10-14

**Authors:** K. Ian White, Yousuf A. Khan, Kangqiang Qiu, Ashwin Balaji, Sergio Couoh-Cardel, Luis Esquivies, Richard A. Pfuetzner, Jiajie Diao, Axel T. Brunger

## Abstract

Membrane fusion is driven by SNARE complex formation across cellular contexts, including vesicle fusion during synaptic transmission. Multiple proteins organize trans-SNARE complex assembly and priming, leading to fusion. One target membrane SNARE, syntaxin, forms nanodomains at the active zone, and another, SNAP-25, enters non-fusogenic complexes with it. Here, we show that the AAA+ protein NSF (N-ethylmaleimide sensitive factor) and SNAP (soluble NSF attachment protein) must act prior to fusion. We show that syntaxin clusters are conserved, that NSF colocalizes with them, and characterize SNARE populations within and near these clusters using cryo-EM. Supercomplexes of NSF, α-SNAP, and either a syntaxin tetramer or two binary complexes of syntaxin—SNAP-25 reveal atomic details of SNARE processing and show how sequential ATP hydrolysis drives disassembly. These results suggest a functional role for syntaxin clusters as reservoirs and a corresponding role for NSF in syntaxin liberation and SNARE protein quality control preceding fusion.

## Full Text Availability

The license terms selected by the author(s) for this preprint version do not permit archiving in PMC. The full text is available from the preprint server.

