# Supplementary material for: Pre-fusion AAA+ remodeling of target-SNARE protein complexes enables synaptic transmission
Source: bioRxiv. 2024 Oct 12:2024.10.11.617886. Preprint. [Version 1] doi: 10.1101/2024.10.11.617886 (PMC11482947; doi:10.1101/2024.10.11.617886)
Supplement: Supplementary file 1 [file media-1.pdf]

Supplemental Information

Pre-fusion AAA+ remodeling of target-SNARE protein complexes enables synaptic transmission

K. Ian White[1,2,3,4,5,**], Yousuf A. Khan[1,2,3,4,5], Kangqiang Qiu[6], Ashwin Balaji[7], Sergio Couoh-Cardel[1,2,3,4,5], Luis Esquivies[1,2,3,4,5], Richard A. Pfuetzner[1,2,3,4,5], Jiajie Diao[6], Axel T. Brunger[1,2,3,4,5,8,*]

[1]Department of Molecular and Cellular Physiology, Stanford University, Stanford, California, United States
[2]Department of Neurology and Neurological Sciences, Stanford University, Stanford, California, United States
[3]Department of Structural Biology, Stanford University, Stanford, California, United States
[4]Department of Photon Science, Stanford University, Stanford, California, United States
[5]Howard Hughes Medical Institute, Stanford University, Stanford, California, United States
[6]Department of Cancer Biology, University of Cincinnati College of Medicine, Cincinnati, United States
[7]Department of Chemistry, Stanford University, Stanford, California, United States
[8]Lead contact

[7]Lead contact

[*]Correspondence: brunger@stanford.edu
[**]Correspondence: kiwhite@stanford.edu

Table of Contents

Supplemental Information *1*

Supplemental discussion *4*

Comparison with p97 .... **4**

Supplemental references *6*

Supplemental figures *7*

Supplemental Figure 1 .... **8**

Supplemental Figure 2 .... **9**

Supplemental Figure 3 .... **11**

Supplemental Figure 4 .... **13**

Supplemental Figure 5 .... **15**

Supplemental Figure 6 .... **17**

Supplemental Figure 7 .... **19**

Supplemental Figure 8 .... **23**

Supplemental Figure 9 .... **24**

Supplemental Figure 10 .... **26**

Supplemental Figure 11 .... **28**

Supplemental Figure 12 .... **30**

Supplemental Figure 13 .... **32**

Supplemental Table 1 34

Supplemental Table 2 35

Supplemental Movie 1 36

Supplemental discussion

Comparison with p97

While this is the first structural evidence for the ADP•P_i state in NSF, the mechanism is consistent with early biochemical study of myosin[3], which proposed the ADP•P_i state and concerted release of Mg^{2+} and P_i, as well as recent EM studies of the proteasome[4] and the NSF homolog p97[5] under hydrolyzing conditions. Indeed, during the preparation of this manuscript, a similar strategy was employed in the study of p97, albeit in the absence of a protein substrate[5]. Three key results from ref. [5] are of interest for comparison with our results.

First, Shein et al. (ref. [5]) note the presence of two distinct sites for Mg^{2+} and P_i following hydrolysis, termed states A and B. In state A, the P_i engages in a bidentate interaction with the Sensor I residue N348 (N374 in NSF) and lies somewhat coaxial with the ADP phosphates, while Mg^{2+} remains coordinated by the ADP Pβ and p97 Walker A residue T252 (T267 in NSF). In state B, both Pi and Mg^{2+} shift downward, apparently related to rotameric shifts of the first arginine finger R359 (R385 in NSF) and a nearby phenylalanine, F360 (P386 in NSF). Furthermore, the p97 mutation F360P, which would remove this rotameric switching, is found to be hyperactivating. Strikingly, we only observe Mg^{2+} and P_i discretely bound in state A, and a proline residue is present at the position equivalent to F360 in wild-type NSF. This provides additional support to the suggestions made by Shein et al. (ref. [5]) that state B is a second transient state that follows state A after ~1 μs as assessed by molecular dynamics simulations, and opens the intriguing idea that the ATPase rate may be tuned by evolution through mutation at position 360/386 in p97/NSF. One might thus expect NSF P386F to have diminished activity and a detectable state B by cryo-EM.

Second, Shein et al. (ref. [5]) note large RMSF values for p97 MD simulations in the ATP state vs. the ADP•P_i state, particularly at the Walker A helix and the NTD-D1 linker. While NSF does not show dramatic flipping of its N-domains with the transition from ATP to ADP, the N-D1 linker and nearby secondary structure elements are nevertheless differentially shifted in a nucleotide-dependent manner that appears propagated through the helix and down to the active site. Completely flipped N-domains are only observed in SNARE-substrate free NSF reconstructions (Fig 5A).

Third, Shein et al. propose an inter-subunit communication pathway in p97. In both p97 and NSF, exactly 11 residues form a semi-structured link between the active sites of two protomers (the "sensor loop"), with the Sensor I motif at the N-terminal end and the first arginine finger at the C-terminal end. The sensor loop represents a plausible pathway by which the Sensor I motif of a given protomer can be modulated allosterically by the presence of nucleotide in the up-ring protomer, but evidence of any conformational transition here is absent in our datasets.

Finally, we do not observe evidence of large-scale conformational change within the loop of NSF corresponding to the so-called intersubunit signaling loop (ISS, NSF residues 359–364) in other

AAA+ proteins, such as Yme1[6,7] and p97[8]. In both proteins, the ISS undergoes a dramatic, nucleotide-dependent transition, in which it is engaged in *trans* in the presence of up-ring ATP, and in *cis* in the presence of up-ring ADP. We observe local changes to the interprotomer interface accompanying P_i release in NSF. However, these changes are limited to small-scale rotameric shifts; no dramatic conformational change is observed, even in the substrate-free, ADP-bound protomers. Given the high degree of sequence divergence in this region, the structural mechanism seen in Yme1 and p97 is likely not employed by NSF.

Supplemental references

1. Khan, Y. A., White, K. I. & Brunger, A. T. The AAA+ superfamily: a review of the structural and mechanistic principles of these molecular machines. *Crit. Rev. Biochem. Mol. Biol.* **57**, 156–187 (2022).
2. Punjani, A. & Fleet, D. J. 3D variability analysis: Resolving continuous flexibility and discrete heterogeneity from single particle cryo-EM. *J. Struct. Biol.* **213**, 107702 (2021).
3. Taylor, E. W., Lymn, R. W. & Moll, G. Myosin-product complex and its effect on the steady-state rate of nucleoside triphosphate hydrolysis. *Biochemistry* **9**, 2984–2991 (1970).
4. de la Peña, A. H., Goodall, E. A., Gates, S. N., Lander, G. C. & Martin, A. Substrate-engaged 26S proteasome structures reveal mechanisms for ATP-hydrolysis–driven translocation. *Science* **362**, eaav0725 (2018).
5. Shein, M. *et al.* Characterizing ATP processing by the AAA+ protein p97 at the atomic level. *Nat. Chem.* **16**, 363–372 (2024).
6. Augustin, S. *et al.* An Intersubunit Signaling Network Coordinates ATP Hydrolysis by m-AAA Proteases. *Mol. Cell* **35**, 574–585 (2009).
7. Puchades, C. *et al.* Structure of the mitochondrial inner membrane AAA+ protease YME1 gives insight into substrate processing. *Science* **358**, eaao0464 (2017).
8. Pan, M. *et al.* Mechanistic insight into substrate processing and allosteric inhibition of human p97. *Nat. Struct. Mol. Biol.* **28**, 614–625 (2021).
9. Ernst, J. A. & Brunger, A. T. High Resolution Structure, Stability, and Synaptotagmin Binding of a Truncated Neuronal SNARE Complex *. *J. Biol. Chem.* **278**, 8630–8636 (2003).
10. Misura, K. M. S., Gonzalez, L. C., May, A. P., Scheller, R. H. & Weis, W. I. Crystal Structure and Biophysical Properties of a Complex between the N-terminal SNARE Region of SNAP25 and Syntaxin 1a *. *J. Biol. Chem.* **276**, 41301–41309 (2001).
11. Goddard, T. D. *et al.* UCSF ChimeraX: Meeting modern challenges in visualization and analysis. *Protein Sci.* **27**, 14–25 (2018).
12. Margittai, M., Fasshauer, D., Pabst, S., Jahn, R. & Langen, R. Homo- and Heterooligomeric SNARE Complexes Studied by Site-directed Spin Labeling *. *J. Biol. Chem.* **276**, 13169–13177 (2001).
13. Punjani, A., Rubinstein, J. L., Fleet, D. J. & Brubaker, M. A. cryoSPARC: algorithms for rapid unsupervised cryo-EM structure determination. *Nat. Methods* **14**, 290–296 (2017).

Supplemental figures

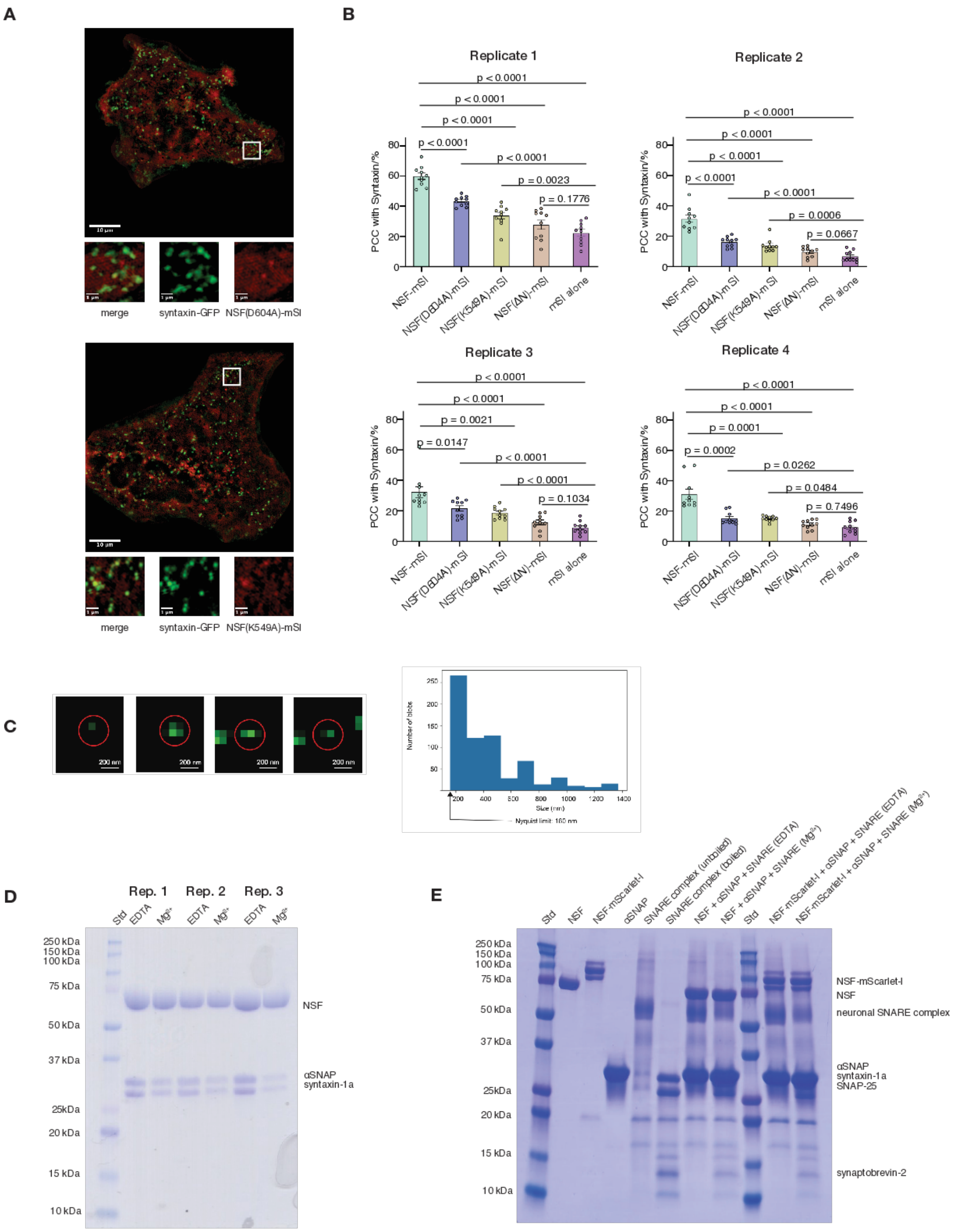

Supplemental Figure 1 | Replicates and supporting materials for syntaxin colocalization (related to Figure 1)

A. Colocalization between syntaxin-GFP nanodomains and two NSF mutants, NSF(D604A)-mScarlet-I (PCC = 43% ± 1%, mean ± SEM) and NSF(K549A)-mScarlet-I (PCC = 33% ± 2%, mean ± SEM). NSF(D604A) is hydrolysis defective, while NSF(K549A) cannot bind nucleotide and thus fails to form a functional hexamer. **B.** Colocalization data and significance tests for four replicate imaging sessions of PC12 cells transfected with each of five different constructs. **C.** Size analysis of 20 SIM images of budding *S. cerevisiae* reveals discrete cluster sizes approaching the Nyquist limit; clusters are likely smaller. **D.** SDS-PAGE result from which densitometry presented in Fig. 1E is derived. **E.** NSF-mScarlet-I retains its ability to disassemble the neuronal SNARE complex as determined by an electrophoretic mobility shift assay (EMSA). Individual components (NSF, NSF-mScarlet-I, ɑ-SNAP, the neuronal SNARE complex) were purified independently (lanes 2–6) and mixed. Then, either EDTA or Mg^{2+} were added as a control, or to initiate the disassembly of the SNARE complex, respectively (NSF, lanes 7 and 8; NSF-mScarlet-I, lanes 10 and 11). Note that the neuronal SNARE complex is SDS and heat resistant, and it runs as a single band (lane 5) unless boiled (lane 6) before SDS-PAGE; lanes 5 and 6 are thus fully assembled or disassembled controls. Comparison of EDTA and Mg^{2+} lanes for either form of NSF shows the appearance of lower molecular weight species matching the boiled SNARE complex control, confirming the disassembly of the SNARE complex by each. NSF-mScarlet-I is thus expected to be hexameric and disassembly-competent in the cellular imaging experiments.

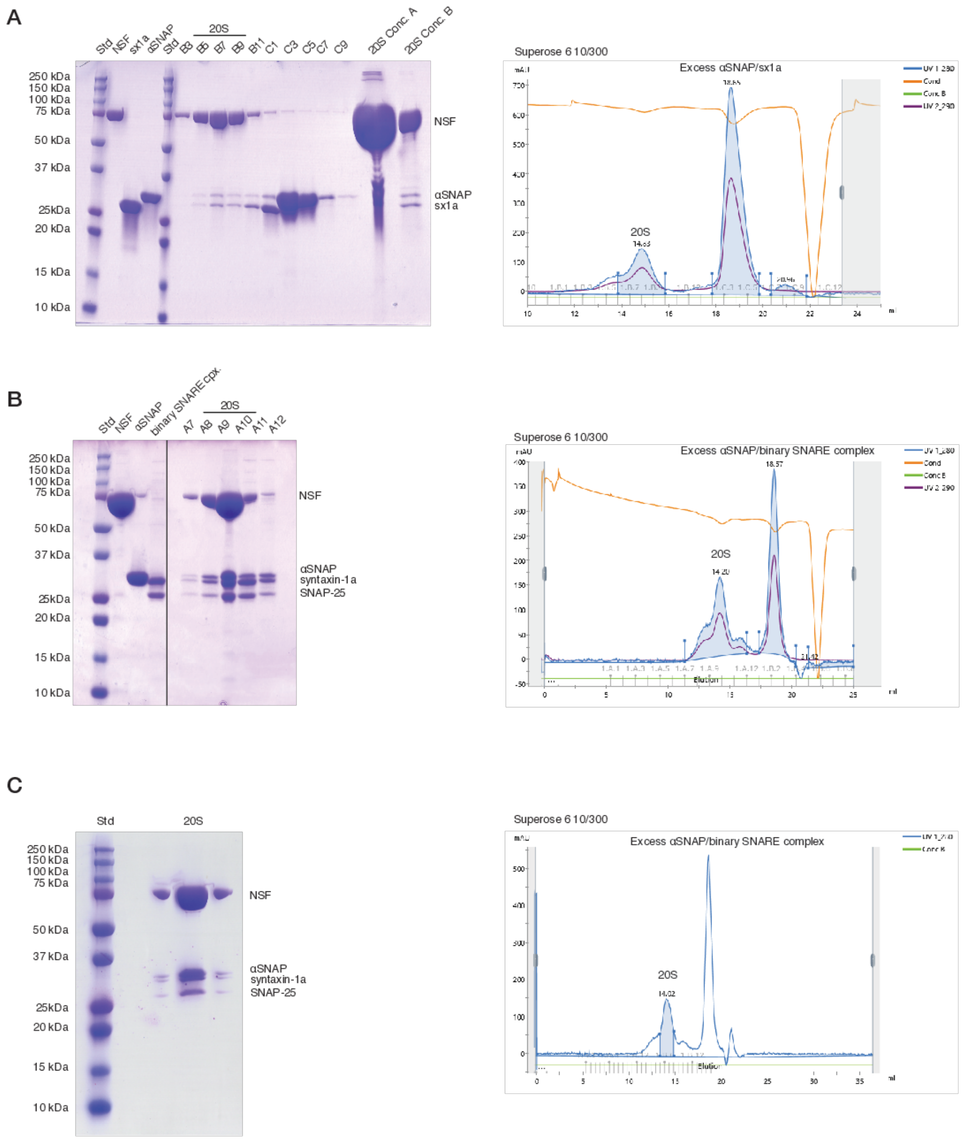

Supplemental Figure 2 | Purification of various complexes for cryo-EM SPA (related to Figures 2–4)

NSF, the specified SNARE complexes, and α-SNAP were each purified recombinantly from *E. coli*, mixed with a 1:5:30 ratio (syntaxin tetramer) or a 1:3:15 ratio (binary complex) under non-hydrolyzing conditions, respectively, and subjected to SEC using a Superose 6 10/300 column. The specified complexes typically eluted with a retention volume of ~14.5 mL and were concentrated to ~15 mg/mL before freezing for cryo-EM. SDS-PAGE was subsequently used to verify the presence of all proteins in the complex. **A.** SDS-PAGE and SEC of the sx20S complex

comprised of NSF, α-SNAP, and soluble syntaxin. EDTA was included throughout to chelate any divalent cation. **B.** SDS-PAGE and SEC of the 21bin20S complex comprised of NSF, α-SNAP, and the syntaxin—SNAP-25 complex. It comprises two soluble syntaxin molecules and one full-length SNAP-25 (2:1 complex). EDTA was included throughout to chelate any divalent cation. **C.** SDS-PAGE and SEC of the 21bin20S complex comprised of NSF, α-SNAP, and the syntaxin—SNAP-25 complex. This stage consists of two soluble syntaxin molecules and one full-length SNAP-25. As in the case of the preparation under non-hydrolyzing conditions, divalent cation was not added to the SEC buffer. However, EDTA was not included, and purification was performed quickly. Following gel filtration and concentration, $MgCl_2$ was added to start the disassembly reaction, converting the sample to the 2:2 complex.

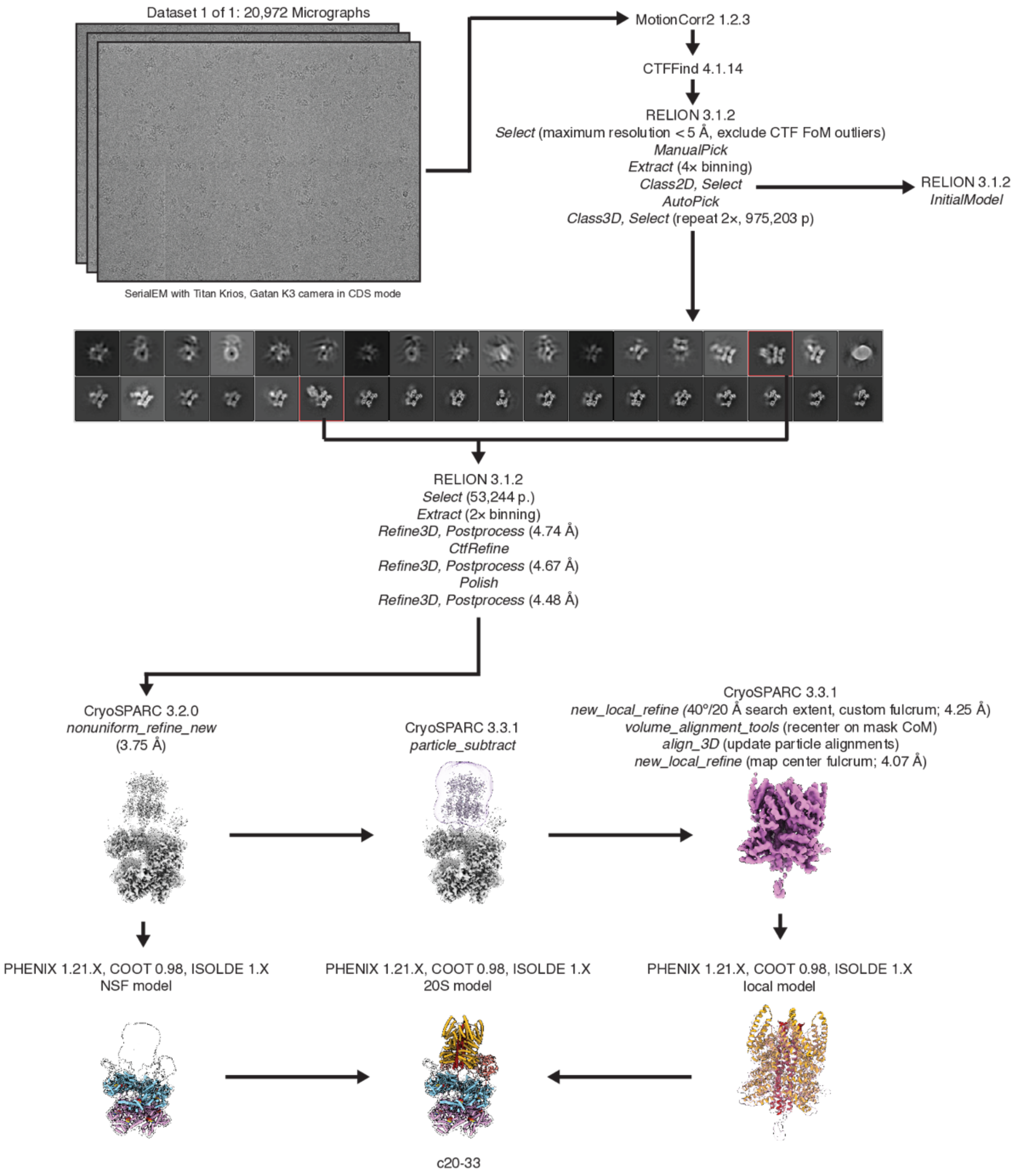

Supplemental Figure 3 | Cryo-EM SPA data collection and processing workflow for the sx20S complex (Related to Figure 2)

A single sx20S cryo-EM dataset frozen under non-hydrolyzing conditions and comprised of 20,972 micrographs was collected and processed, yielding a single sx20S class with 53,244 particles. The remaining classes were substrate-free and were discarded; this is not due to the disassembly of the syntaxin tetramer, but rather the inability to fully separate NSF from the

sx20S complex by SEC. The sx20S particles produced a single sx20S reconstruction with an overall resolution of 3.75 Å. Modeling of the NSF D1 and D2 rings was performed with this reconstruction. Subsequently, this reconstruction was used as the basis for signal subtraction and local reconstruction of the α-SNAP—syntaxin tetramer subcomplex, and the α-SNAP—syntaxin tetramer subcomplex model was built with this reconstruction and then docked back into the full sx20S reconstruction. See Fig. S9A and Methods for further details.

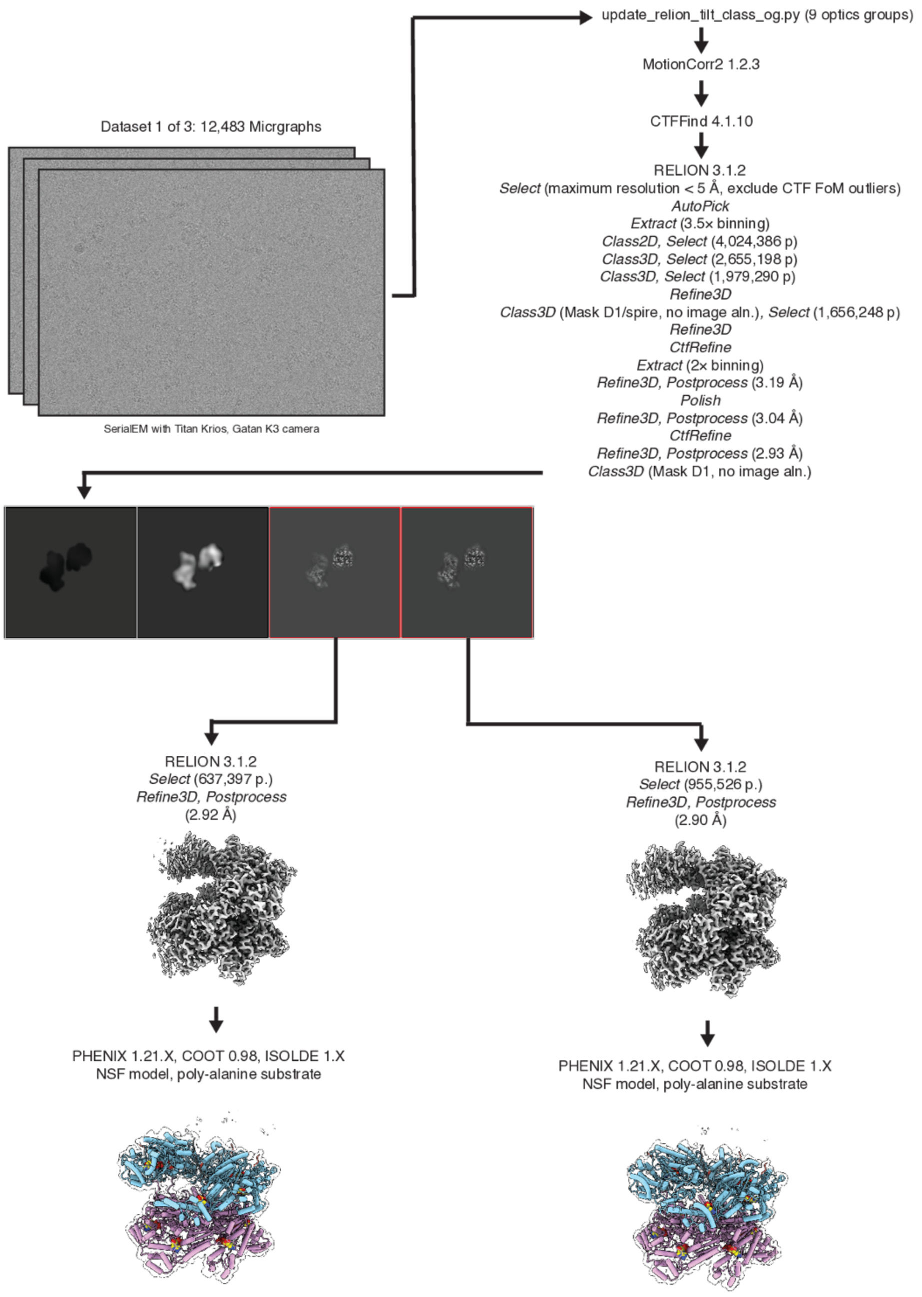

Supplemental Figure 4 | Cryo-EM SPA data collection and processing workflow for the high-resolution NSF D1/D2 reconstructions under non-hydrolyzing conditions (Related to Figure 3)

A single cryo-EM SPA dataset from 21bin20S sample frozen under non-hydrolyzing conditions and comprised of 12,483 micrographs was collected and processed, yielding two classes (class 4-3-5-3, engaged F protomer, 2.92 Å, and class 4-3-5-4, disengaged F protomer, 2.90 Å) with near-atomic resolution D1 and D2 reconstructions. The data processing strategy averages out spire density and thus precludes α-SNAP—SNARE subcomplex modeling, so substrate in the D1 pores of each class is modeled as a poly-alanine chain. Due to their high quality, these models served as starting points for all other classes reported herein. See Fig. S9 and Methods for further details.

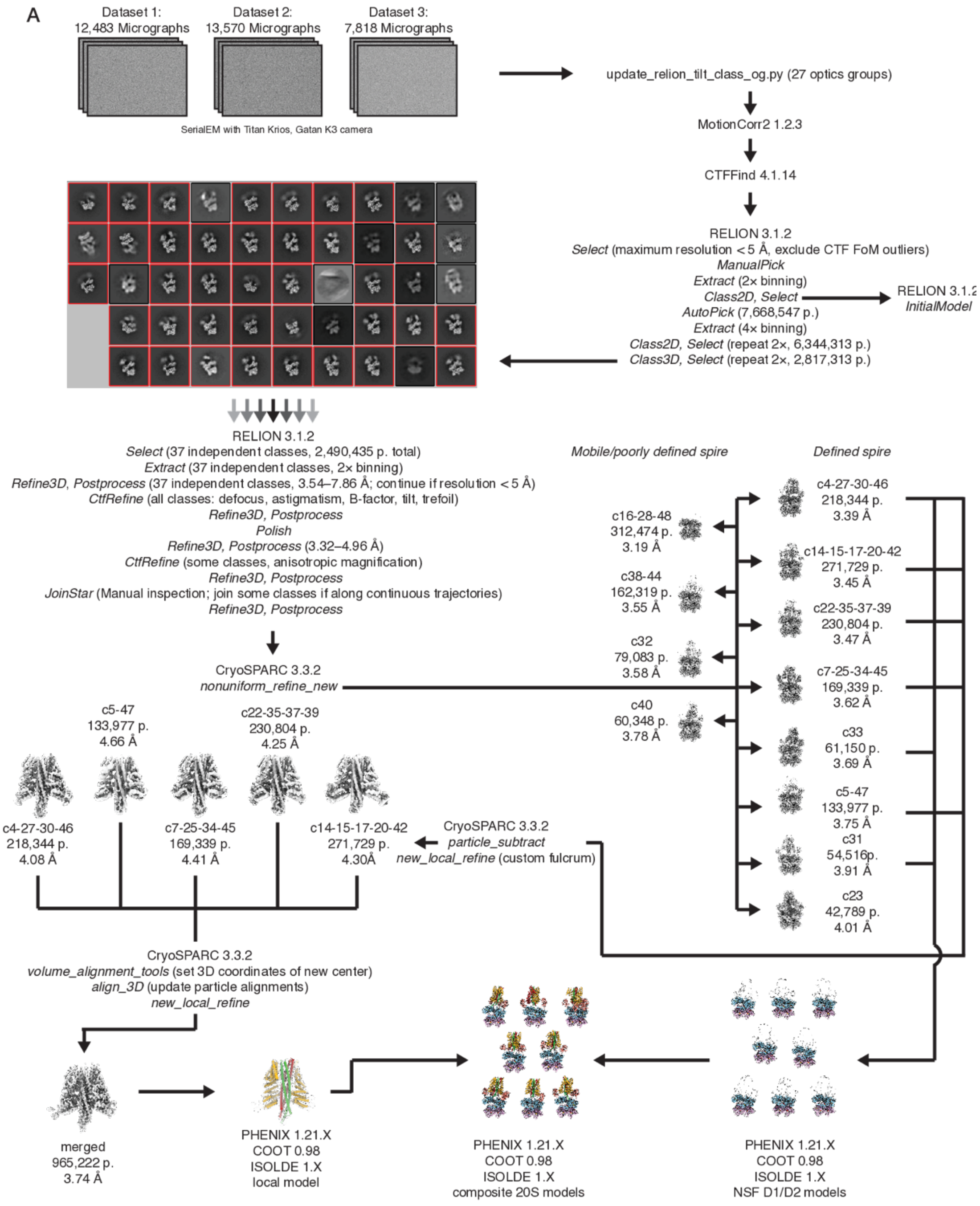

Supplemental Figure 5 | Cryo-EM SPA data collection and processing workflow for the 21bin20S complex under non-hydrolyzing conditions (Related to Figure 3)

Three cryo-EM SPA datasets collected from the 21bin20S sample frozen under non-hydrolyzing conditions, including the dataset described in Fig. S4, were combined for a total of 33,871 micrographs. Ultimately, nearly 2.5 million particles were classified into 37 independent classes; classes along continuous trajectories of conformational change were merged, yielding twelve total classes with resolutions ranging from 3.19–4.01 Å. Eight classes with at least partially ordered spire density were carried forward to model the NSF D1 and D2 ATPase rings; all show substrate-engaged F protomers and differ largely based on spire pose. Signal subtraction and local refinement of the α-SNAP—SNARE subcomplex of five of these classes having more than 100,000 particles was then performed. Then the particles from these local reconstructions were subsequently aligned to yield one final merged reconstruction with 965,222 particles and a final resolution of 3.74 Å. A model was built into the α-SNAP—2:1 binary SNARE subcomplex and subsequently docked back into the full 21bin20S reconstructions, yielding eight total 21bin20S models. See Fig. S9A and Methods for more details.

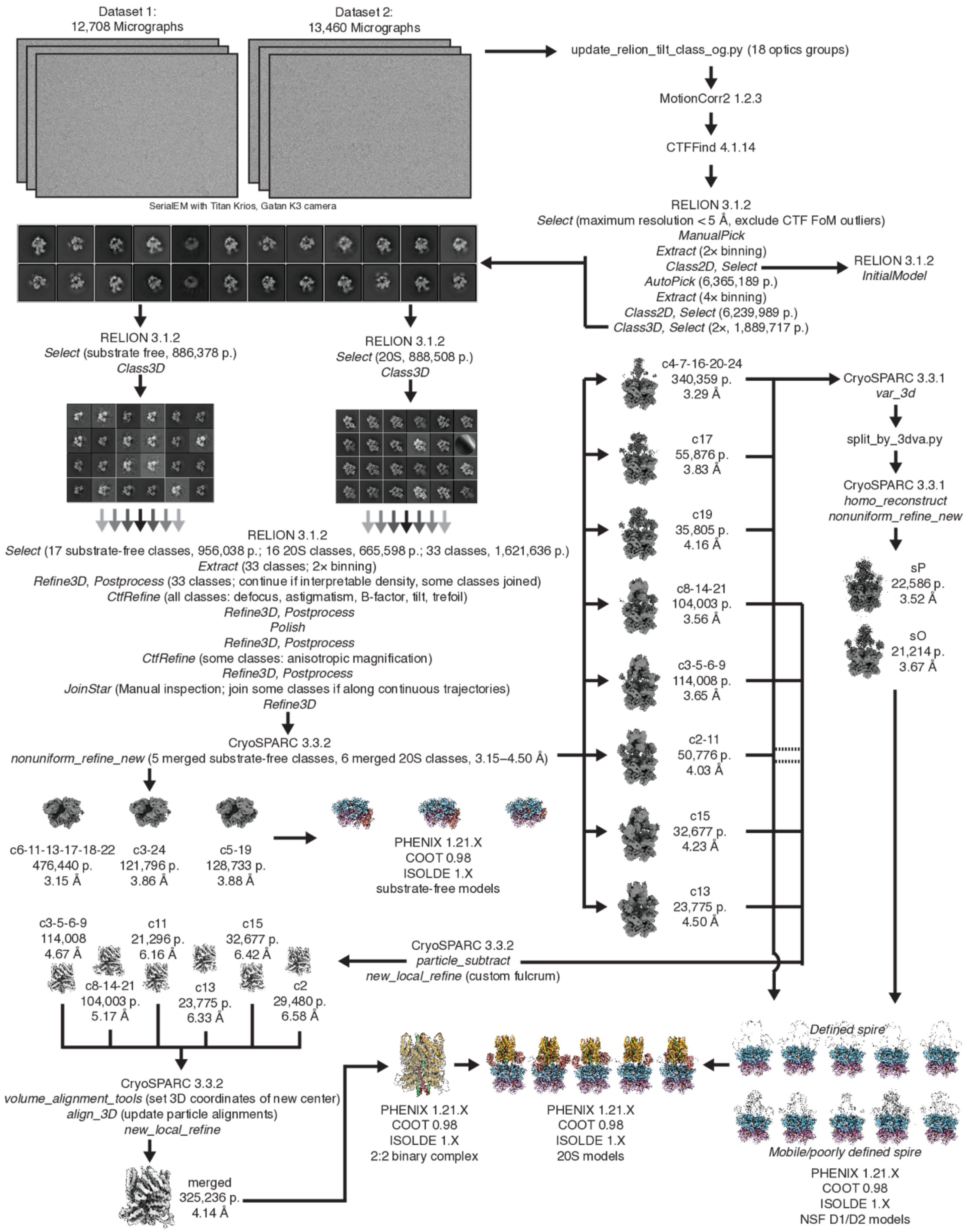

Supplemental Figure 6 | Cryo-EM SPA data collection and processing workflow for the 22bin20S complex under hydrolyzing conditions (Related to Figure 4)

Two cryo-EM SPA datasets were collected from 22bin20S samples frozen under hydrolyzing conditions ~1 min after adding a mixture containing Mg^{2+} and ATP regeneration components creatine phosphate and creatine kinase. Together, these data collections yielded 26,168 total micrographs. 3D classification of nearly two million particles yielded classes falling into two categories—22bin20S, with ordered α-SNAP—SNARE subcomplex density and density in the NSF D1 ATPase pore, or substrate-free NSF, with no density in the pore and a distinctive double split in both the D1 and D2 rings. After splitting these ~1.6 million particles into roughly equal sets, performing further independent 3D classifications, and merging classes with continuous disorder, a total of eight 22bin20S classes with ~600,000 particles (3.29–4.50 Å) and three substrate-free NSF classes with ~1 million particles (3.15–3.88 Å) were carried forward. 3DVA was performed on the largest 22bin20S class (class 4-7-16-20-24), which revealed substantial conformational change in the NSF D1 ring over the first mode. Around 20,000 particles from the tails of the 3DVA distribution were thus used to produce two additional 22bin20S reconstructions, for a total of ten. Models were then built into the three substrate-free NSF reconstructions and the ten 22bin20S reconstructions. Signal subtraction and local refinement of the α-SNAP—SNARE subcomplex of six 22bin20S classes was then performed. Then, the particles from these local reconstructions were subsequently aligned to yield one final merged reconstruction with 325,236 particles and a final resolution of 4.14 Å. A model was built into the α-SNAP—2:2 binary SNARE subcomplex and subsequently docked back into the full 22bin20S reconstructions with well-defined spires, yielding five total 22bin20S models. See Fig. S9A and Methods for more details.

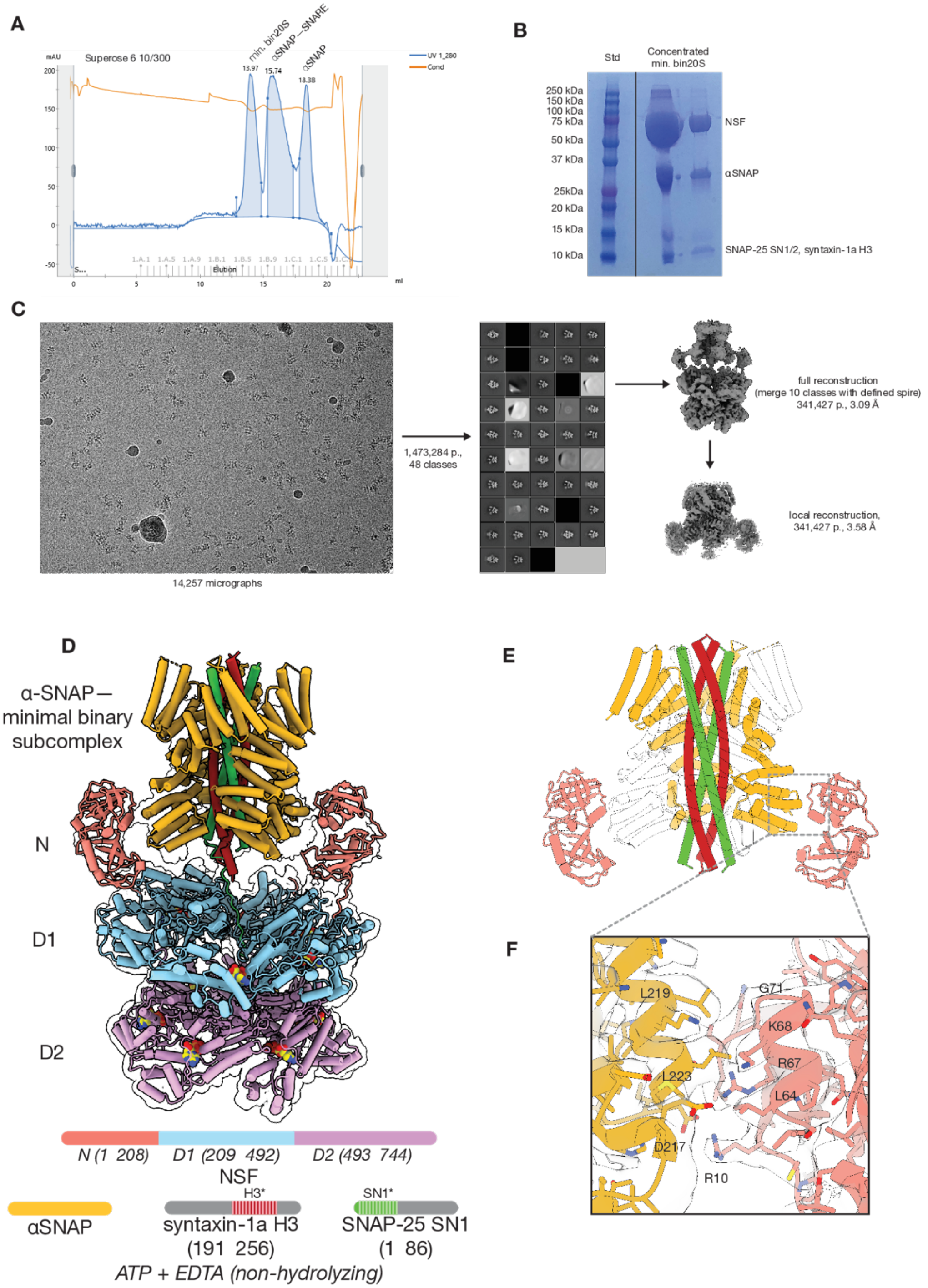

Supplemental Figure 7 | NSF—α-SNAP—2:2 minimal syntaxin—SNAP-25 complex structure

A. NSF, α-SNAP, SNAP-25A SN1 SNARE domain (residues -2–83), SNAP-25A SN2 SNARE domain (residues 141–204), and syntaxin-1a H3 SNARE domain were each purified recombinantly from *E. coli*, mixed with a 1:5:20 ratio under non-hydrolyzing conditions, respectively, and subjected to size exclusion chromatography (SEC) using a Superose 6 10/300 column. The minimal bin20S complex eluted with a retention volume of ~14 mL and was concentrated to ~20 mg/mL before freezing for cryo-EM. SDS-PAGE was subsequently used to verify the presence of the 20S complex. **B.** A single minimal bin20S cryo-EM dataset frozen under non-hydrolyzing conditions and comprised of 14,257 micrographs was collected and processed. As the primary goal of this collection was to identify the stoichiometry of the SNARE substrate in the spire, all classes with defined spire density were merged into a single minimal bin20S superclass with 341,427 particles. These particles were used to reconstruct density for the minimal bin20S complex with a resolution of 3.09 Å. Modeling of the NSF D1 and D2 rings was performed with this reconstruction. Subsequently, this reconstruction was used as the basis for signal subtraction and local reconstruction of the NSF N-domain—α-SNAP—binary SNARE subcomplex to 3.86 Å resolution. **C.** The overall architecture of the minimal bin20S supercomplex. **D.** The structure of the minimal 2:2 syntaxin—SNAP-25 subcomplex (class #27), with two of four α-SNAP molecules hidden for clarity. This configuration matches a previously reported crystal structure of the binary complex[10] and the full-length bin20S SNARE subcomplex observed by cryo-EM in this work (Fig. 4). While the SNAP-25 SN2 SNARE domain was copurified with the other two SNARE domains, it was not present in the EM density. **E.** The local reconstruction of the α-SNAP—minimal 2:2 syntaxin—SNAP-25 subcomplex (class #28) reveals new detail into the primary interaction between the N-domains and α-SNAP. This interface is characterized by hydrophobic α-SNAP sidechains L219 and L223 packing near NSF N-domain residues 64–71; both sides of this interaction are α-helical. Nearby, α-SNAP D217 forms a salt bridge with NSF N-domain R10.

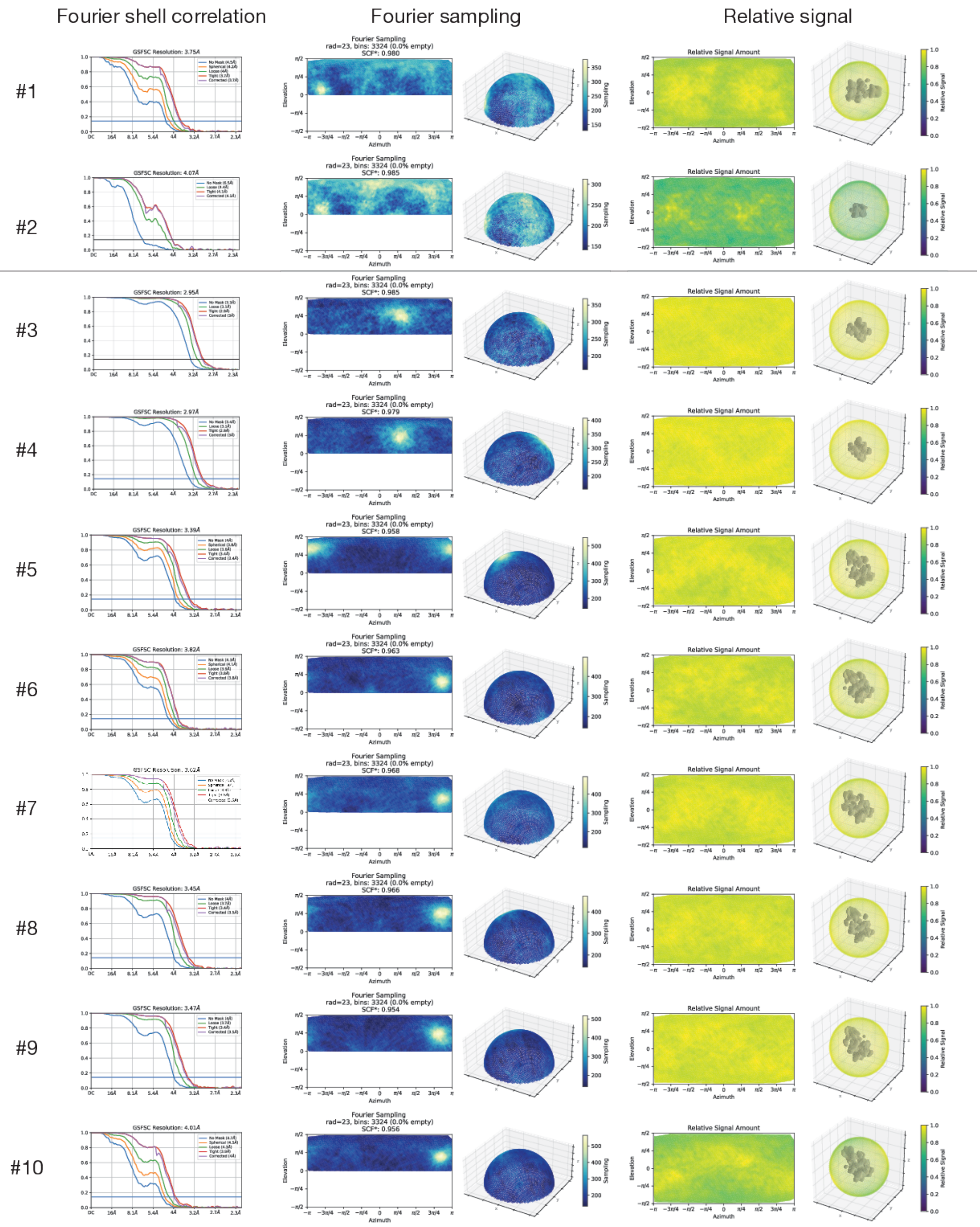
Fourier shell correlation
Fourier sampling
Relative signal
#1
#2
#3
#4
#5
#6
#7
#8
#9
#10

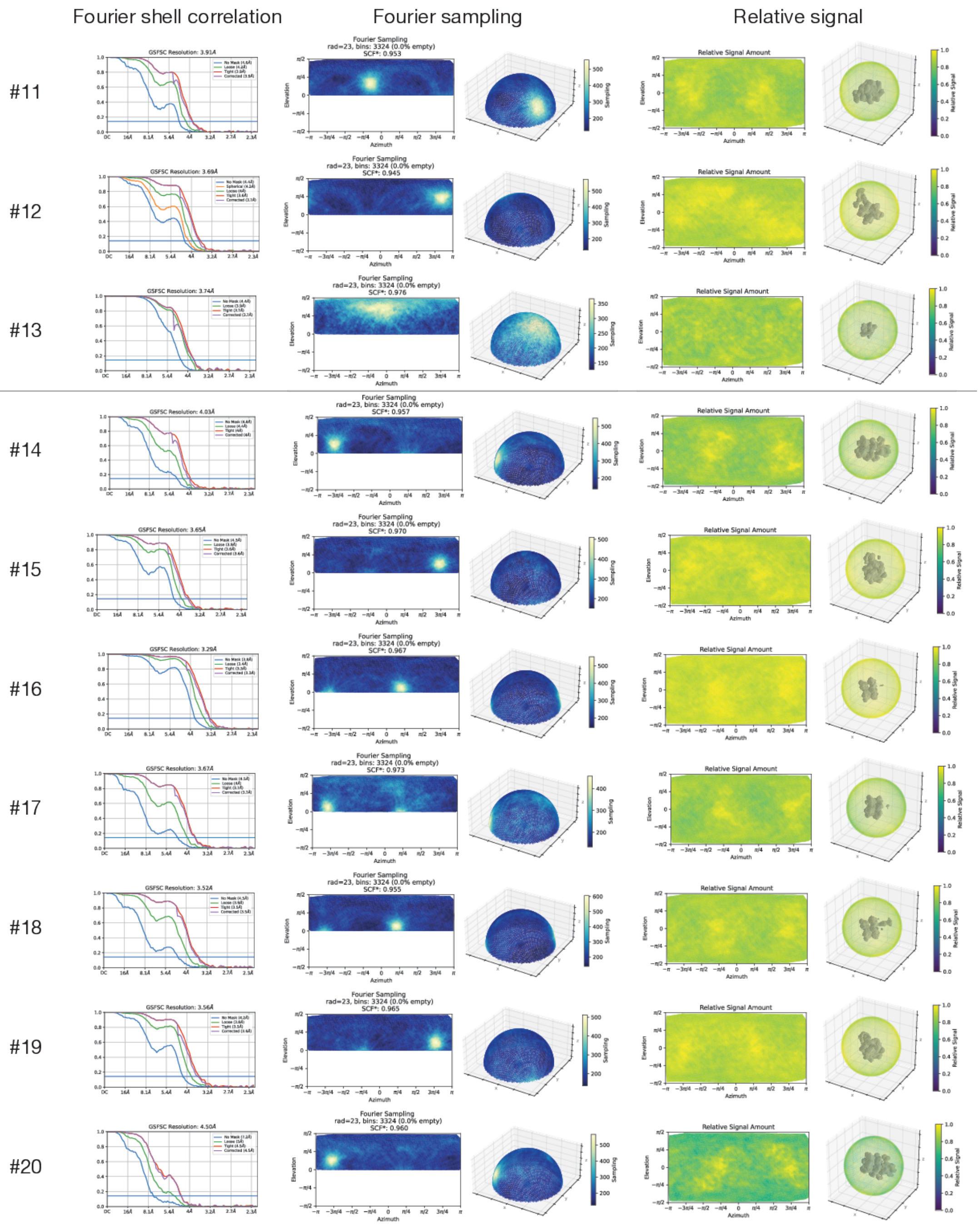
Fourier shell correlation
Fourier sampling
Relative signal
#11
#12
#13
#14
#15
#16
#17
#18
#19
#20

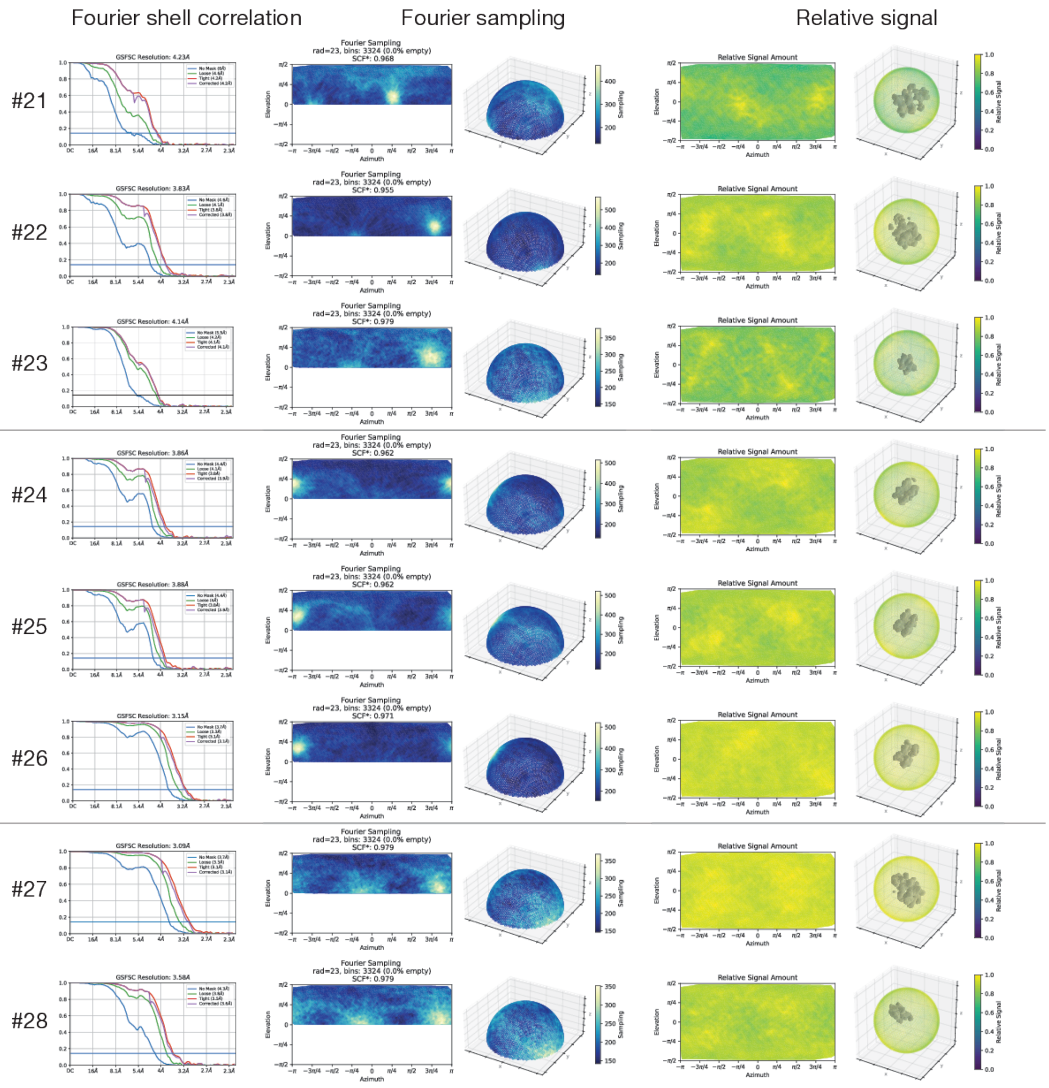

Supplemental Figure 8 | Fourier shell correlation (FSC) curves, Fourier sampling, and relative signal for all reconstructions.

See Tables S1–2 for corresponding details.

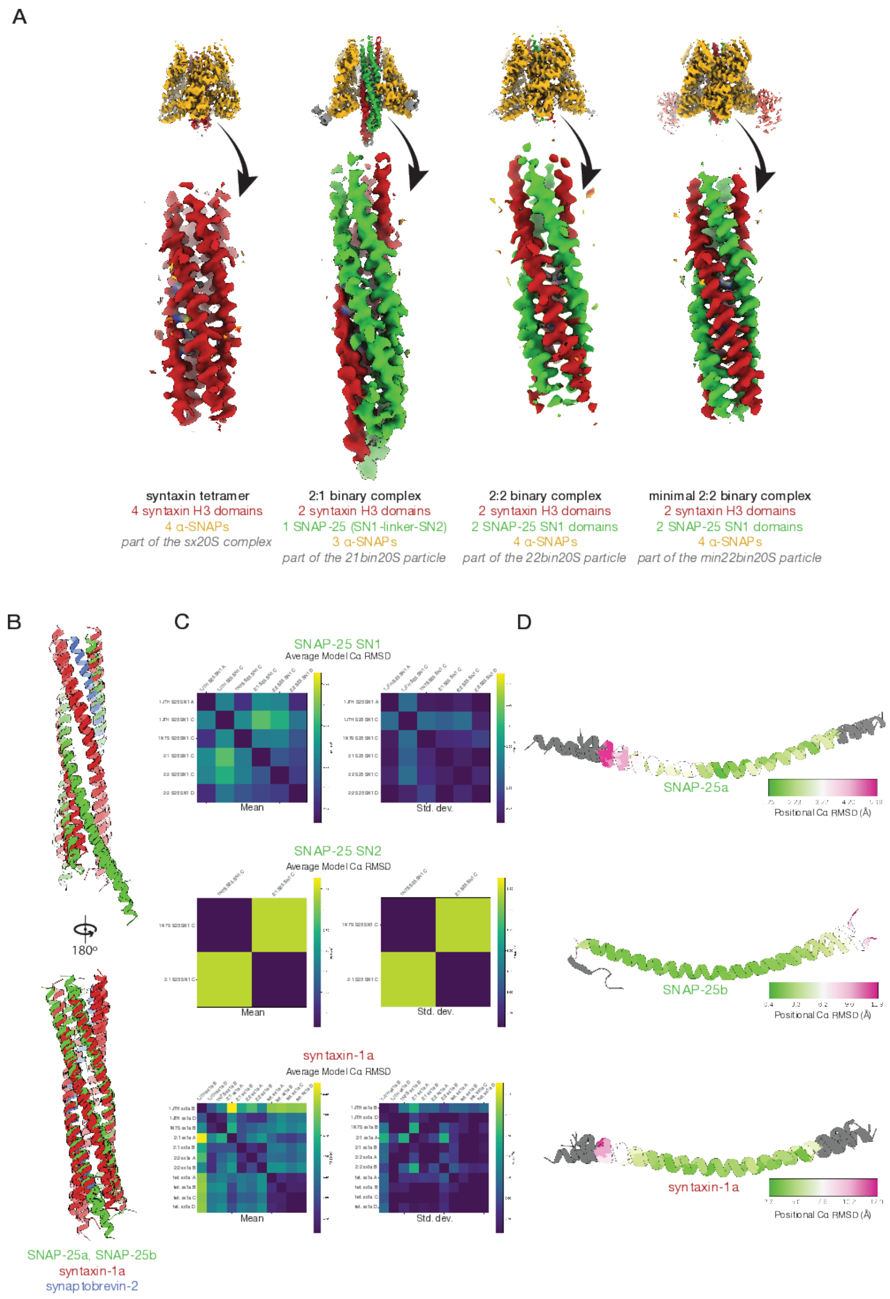

Supplemental Figure 9 | Reconstruction and comparison of SNARE complexes and sub-complexes

A. Signal subtraction and local refinement in CryoSPARC[13] produced high-quality reconstructions of four different α-SNAP—SNARE subcomplexes. In each case, the density of the SNARE proteins in the core of the subcomplex was sufficient to assign the SNARE domain identity, directionality, and register. **B.** Crystal structures of the neuronal ternary SNARE complex (PDB ID 1N7S[9]) and the 2:2 syntaxin—SNAP-25 complex (PDB ID 1JTH[10]) were

superimposed using UCSF ChimeraX[11] with cryo-EM structures from this work of the tetrameric syntaxin H3 sub-complex, the 2:1 syntaxin—SNAP-25 sub-complex, and the 2:2 syntaxin—SNAP-25 sub-complex within the bin20S complex. These structures show similar quaternary structure, maintaining the same register and similar twist in the central SNARE layers (see also Figs. 2–4D). The SNARE complexes and sub-complexes within their respective 20S complexes are somewhat untwisted at the N- and C-terminal ends relative to the crystal structures of the SNARE complexes alone, likely due to a combination of the absence of crystal packing as well as interactions with α-SNAPs and NSF. The similarity of this syntaxin—SNAP-25 to the ternary SNARE complex is also consistent with previous electron paramagnetic resonance (EPR) spectroscopy studies[12]. **C.** Quantitative comparison of individual SNARE domain ternary structure using the custom script *compare_snares.py*. Each pixel in a given pair of matrices represents the mean Cα RMSD (Å, left) and corresponding standard deviation (right) between two instances of a given SNARE domain in two different structures. Only Cα atoms shared by all structures were considered. The same color scale is preserved across all comparisons. The mean Cα RMSD generally varies between 1–2 Å depending on the specific pair. **D.** Individual Cα RMSDs mapped to their corresponding SNARE domain structures, aligned. RMSDs were calculated with *compare_snares.py* and visualized with UCSF ChimeraX[11]. Core regions of the complexes are structurally similar, but unwinding near the SNARE domain termini leads to larger deviations, particularly near the N-termini where NSF engages one of the N-terminal segments leading into a SNARE domain (SNAP-25 or syntaxin).

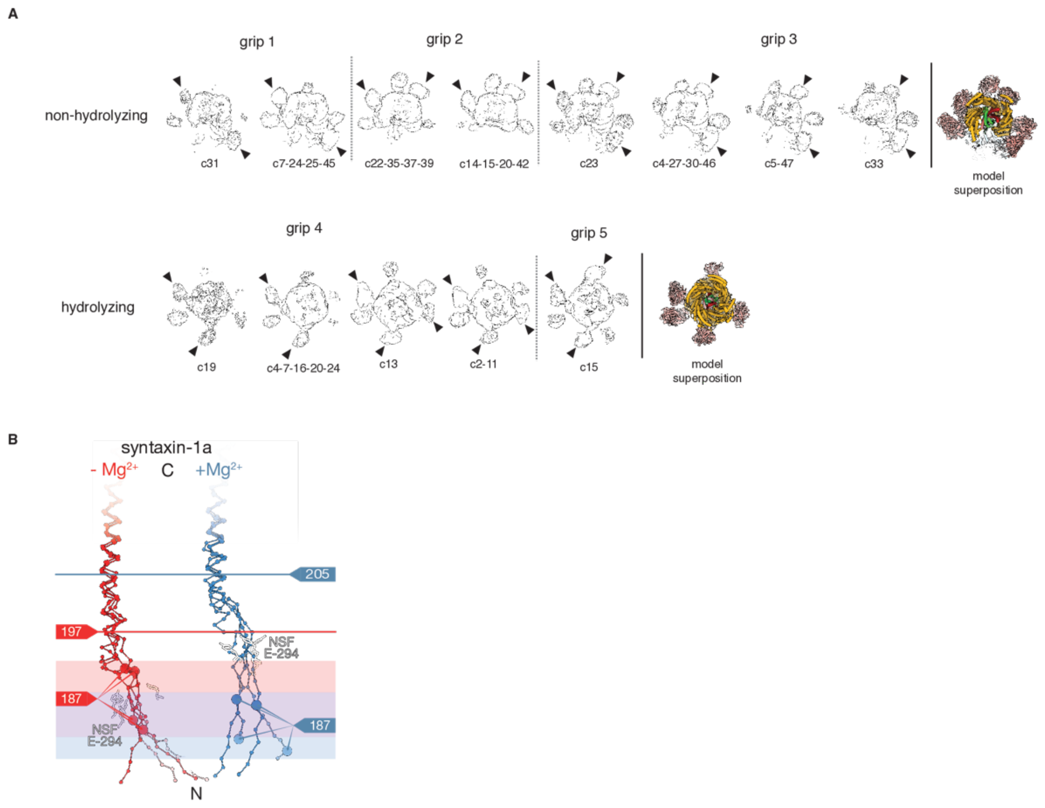

Supplemental Figure 10 | Multimodal engagement of SNARE substrate by NSF

A. The α-SNAP—SNARE subcomplex is engaged by the NSF N domains. The orientation of the subcomplex relative to the NSF D1 ring split, the tilt of the subcomplex relative to the D1 ring, and the specific NSF N-domains engaged to the subcomplex are major determinants of particle class identity in different datasets. Three distinctive "grips" are observed in the case of the classes with the 2:1 syntaxin—SNAP-25 complex identified under non-hydrolyzing conditions (top), and two are observed in the case of the classes with the 2:2 syntaxin—SNAP-25 SNARE complex identified under hydrolyzing conditions (bottom). Pairs of N-domains typically bind a given α-SNAP near its C-terminus; arrows indicate the most well-ordered N-domains as assessed by the presence of two-lobed density. The two cases are not directly comparable, as the 2:1 syntaxin—SNAP-25 sub-complex is bound by three α-SNAP molecules, while the 2:2 syntaxin—SNAP-25 sub-complex is bound by four α-SNAP molecules. This difference is likely due to the flexible linker that runs along the 2:1 syntaxin—SNAP-25 sub-complex surface between the SNAP-25 SN1 and SN2 SNARE domains. **B.** The SNARE N-termini are engaged by the NSF D1 pore. Variable syntaxin engagement by NSF between non-hydrolyzing and hydrolyzing classes is observed. Alignment of structures with ordered syntaxin substrates in the

pore of the D1 ring under non-hydrolyzing (red) and hydrolyzing (blue) conditions reveals disruption of the syntaxin H3 domain up to around residues 197 and 205, respectively.

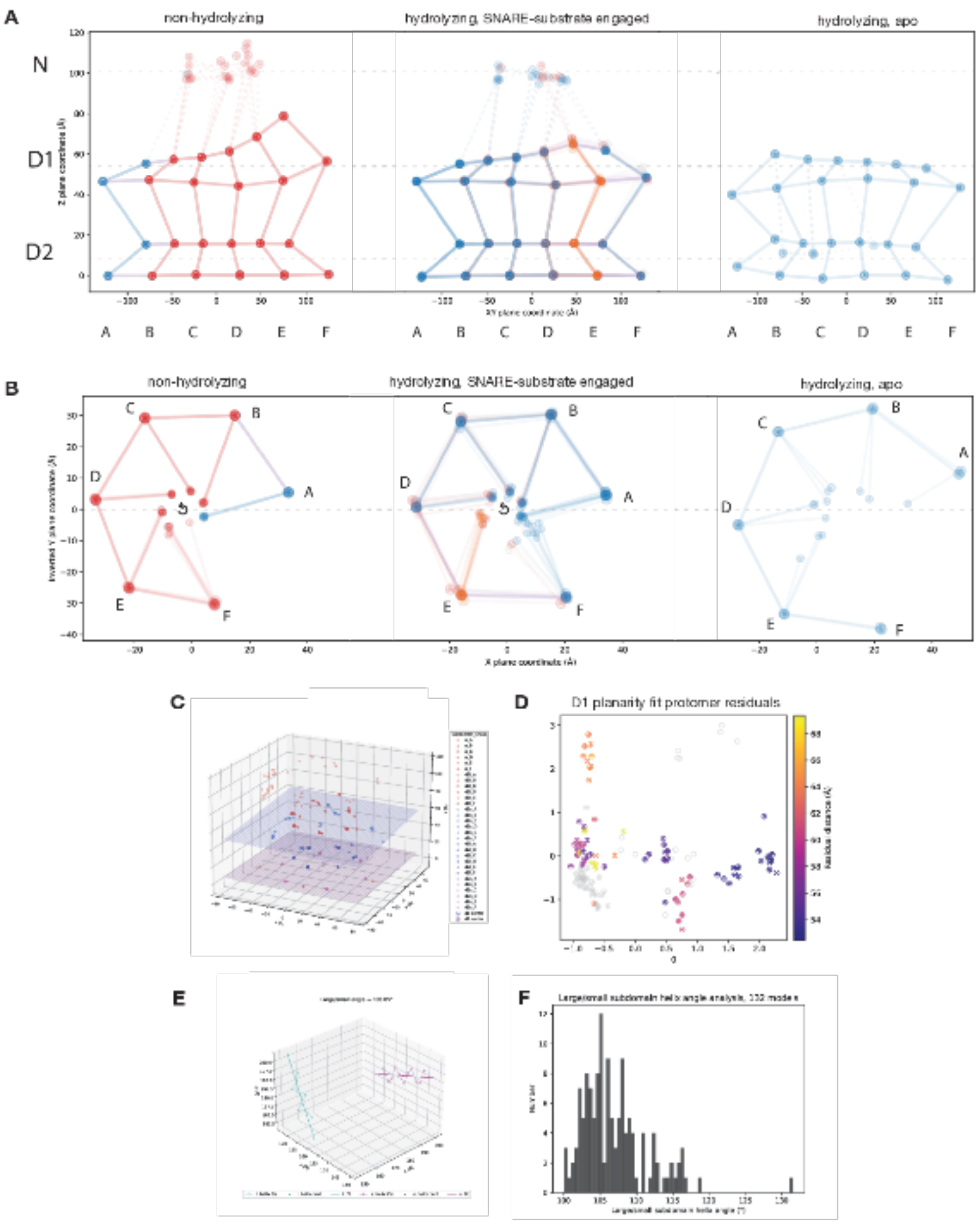

Supplemental Figure 11 | Additional details for NSF D1 and D2 planarity analysis and large-small subdomain angle analysis.

These panels supplement Fig. 6. **A.** Centers of mass for N-domains and the D1 large, D1 small, D2 large, and D2 small subdomains in 3D and planes were fit to the subdomains corresponding

to the D1 and D2 rings (see also **C**). The results are projected into the 2D, with the NSF protomer subdomain centers of mass unrolled into a single plane to illustrate their relative positions. Dashed lines indicate the plane fit. A given protomer is colored by the nucleotide found in its D1 subdomain; ADP-bound protomers are colored blue, ATP-bound protomers are colored red, ATP-Mg^{2+}-bound protomers are colored orange, and nucleotide-free protomers are colored grey. This comparison illustrates the high degree of structural similarity between the D2 rings between datasets, in contrast to the high degree of conformational change in the up-ring protomers of D1. These conformational differences are largely coaxial with the D1 pore. More variation is present in the hydrolyzing dataset than in the non-hydrolyzing dataset. **B.** Projection of D1 protomer centers of mass into the plane of the D1 ring reveals conformational change associated with protomer F rebinding; the non-hydrolyzing structure projection reveals the limits of F protomer disengagement and engagement, while protomers F in the hydrolyzing dataset are more variable and transition between these states. This projection also illustrates how the SNARE substrate-free D1 rings retract from the pore axis. **C.** Raw plane fits and centroids in 3D used to generate **A** and **B**. See Methods for more details. **D.** NSF large subdomain and small subdomain D1 plane fit residuals for a given protomer (substrate-bound, hydrolyzing dataset protomers A–F and non-hydrolyzing protomers A and F) were summed and used to color structures projected into the PCA space (Fig. 6) to illustrate planarity changes along the conformational coordinate associated with hydrolysis. **E.** Example helical axes fits for one of 132 NSF D1 large-small subdomain angle calculations. The smallest angle between vectors defined by these fits is then calculated. **F**. Overall distribution of large-small subdomain angles over 132 protomers from 22 models in the dataset.

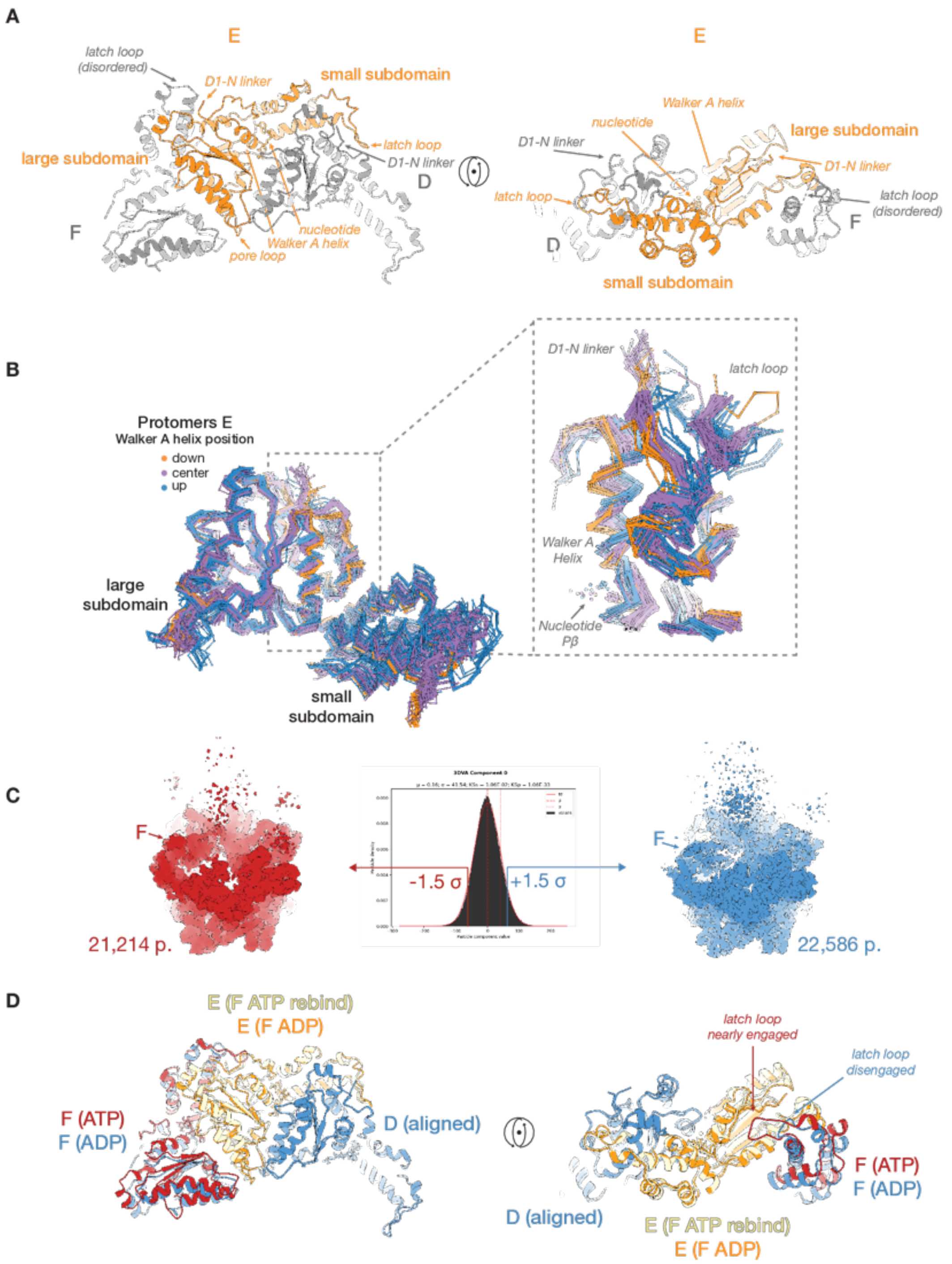

Supplemental Figure 12 | Unique features of protomers E from the hydrolyzing dataset are connected to protomer F rebinding.

These panels are supplemental to those shown in Figs. 5–6. **A.** Two views of a protomer E from the hydrolyzing dataset (colored orange), with neighboring protomers F and D (colored grey). Various notable features are annotated. **B.** All protomers from the hydrolyzing condition, and protomers A and F from the non-hydrolyzing condition are aligned by their D1 large subdomains. SNARE substrate-free models from the hydrolyzing condition are not shown. N-domains and D2 domains are hidden. Protomers E from the hydrolyzing condition show a unique Walker A α-helix conformation (orange models, "down"), in which the helix is shifted down and tilted towards the D1 pore. Most protomers fall into an intermediate state (purple, "center"), except for the protomers A, which occupy a third state (blue, "up"). This is connected to two key features—the N-D1 linker position, for which protomers E have a unique conformation, and the latch loop conformation, which varies from a disengaged and helical state, through an unstructured state, and ultimately to an engaged state. In all but one case, protomers E have a disengaged protomer F up-ring, and thus an intermediate latch loop state. Critically, the engagement of protomer F involves an interaction between the latch loop and the N-D1 linker, which is pulled away from the Walker A helix, allowing it to shift into the center or up states (inset, right). See Fig. 6 for more details. **C.** *CryoSPARC* 3D Variability Analysis (3DVA)[2] captured the transition between the disengaged and engaged protomer F states. 3DVA was run on a large class from the hydrolyzing dataset (see Fig. S6), and particles in the ±1.5 σ tails of the first mode were used to reconstruct the most extreme states along the coordinate. The change in protomer F state, as well as a shift in protomer A towards the split, are evident on inspection of unsharpened maps. **D.** Sub-models showing the states of protomers D, E, and F derived from the density in **C**. The latch loop of protomer F engages protomer E to a degree approaching the state found in the non-hydrolyzing condition (not shown).

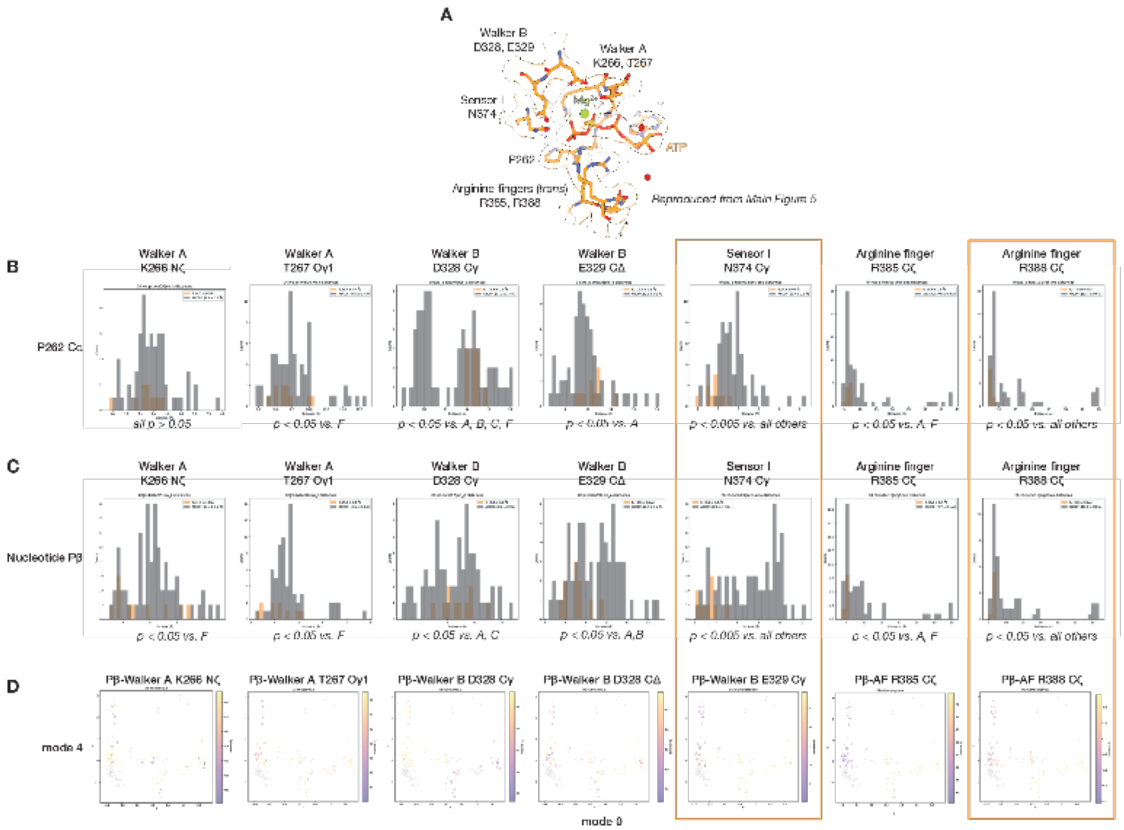

Supplemental Figure 13 | The pre-hydrolysis state of protomers E has several unique active site features.

A. Active site geometry from an NSF protomer E D1 domain. ATP is found in the P-loop, where Walker A residues K266 and T267 coordinate Mg^{2+} and Pγ, respectively. The Sensor I residue N374 coordinates Pγ. The acidic Walker B residues D328 and E329 are oriented towards the nucleotide. The arginine fingers of the down-ring protomer D coordinate the nucleotide phosphates from the opposing direction. **B.** Distance distributions between the P262 Cα and a given active site residue. P262 Cα is a relatively invariant reference atom for measuring distances, as it is well-constrained by local geometry and the presence or absence of Pγ, Pi, or Mg^{2+} in the active site. Protomers E (orange bars) have a significantly closer Sensor I N374 distance to P262 (4.8 ± 0.5 Å vs. 5.7 ± 0.9 overall, $p < 0.005$ for all pairs) than any other protomer (black bars). The terminal carbon of the second arginine finger, R388 Cζ, was also significantly closer in protomers E than in any other protomer (7.5 ± 0.4 Å vs. 13.7 ± 9.6 Å, $p < 0.05$ for all pairs). While not a specific observation to protomer E, the P262 Cα-Walker B D328 Cγ distance distribution is bimodal, with ADP-bound protomers generally having a smaller distance between the two atoms. Welch's *t*-test, assuming unequal variances, was used to assess significance in all cases. **C.** Distance distributions between the Pβ atom of either ATP or ADP of

a given active site residue. Pβ was used as it is found in all nucleotide-bound protomers. The pattern of significant differences between protomers E and protomers A–D and F described for P262 Cα is mostly repeated here, with the Sensor I N374 average distance to Pβ found to be highly significant (6.6 ± 0.4 Å vs. 8.6 ± 1.3, $p < 0.005$ for all pairs). As in **B**, the terminal carbon of the second arginine finger, R388 Cζ, was also significantly closer to Pβ in protomers E than in any other protomer (6.3 ± 0.6 Å vs. 11.6 ± 8.8, $p < 0.05$ for all pairs). Some differences are present in other cases. Aside from specific concerns related to protomers E, many of these distributions also show a bimodal pattern consistent with different nucleotide states. Welch's *t*-test, assuming unequal variances, was used to assess significance in all cases. **D.** Distances from the histograms in **C** mapped to the PCA space comprised of modes 0 and 4 from Fig. 6, enabling comparison between active site residue distances and the different properties outlined there, including HDBSCAN cluster ID, protomer ID, nucleotide state, Walker A helix position, N-D1 linker position, and latch loop engagement.

Extended Data Table 1: Cryo-EM data collection, refinement, and validation statistics

	NSF—αSNAP—syntaxin H3 SNARE domain tetramer (substrate bound, non-hydrolyzing; sx20S)		NSF—αSNAP—2:1 syntaxin:SNAP-25 SNARE complex (substrate bound, non-hydrolyzing; 21bin20S)						
	#1 (c20-33 full) (EMDB-XXXXX) (PDB YYYY)	#2 (c20-33 local) (EMDB-XXXXX) (PDB YYYY)	#3 (c4-3-5-3) (EMDB-XXXXX) (PDB YYYY)	#4 (c4-3-5-4) (EMDB-XXXXX) (PDB YYYY)	#5 (c4-27-30-46) (EMDB-XXXXX) (PDB YYYY)	#6 (c5-47) (EMDB-XXXXX) (PDB YYYY)	#7 (c7-25-34-45) (EMDB-XXXXX) (PDB YYYY)	#8 (c14-15-20-42) (EMDB-XXXXX) (PDB YYYY)	#9 (c22-35-37-39) (EMDB-XXXXX) (PDB YYYY)
**Data collection and processing**									
Microscope, camera	Titan Krios, K3	Titan Krios, K3	Titan Krios, K3	Titan Krios, K3	Titan Krios, K3	Titan Krios, K3	Titan Krios, K3	Titan Krios, K3	Titan Krios, K3
Magnification	22,500×	22,500×	22,500×	22,500×	22,500×	22,500×	22,500×	22,500×	22,500×
Voltage (kV)	300	300	300	300	300	300	300	300	300
Dataset ID(s)	20210903	20210903	20190624	20190624	20190624, 20190919, 20191216	20190624, 20190919, 20191216	20190624, 20190919, 20191216	20190624, 20190919, 20191216	20190624, 20190919, 20191216
Electron exposure (e–/Å²)	27.608	27.608	31.560	31.560	31.560, 36.760, 35.480	31.560, 36.760, 35.480	31.560, 36.760, 35.480	31.560, 36.760, 35.480	31.560, 36.760, 35.480
Defocus range (μm)	-1.0 to -3.0	-1.0 to -3.0	-1.0 to -3.0	-1.0 to -3.0	-1.0 to -3.0	-1.0 to -3.0	-1.0 to -3.0	-1.0 to -3.0	-1.0 to -3.0
Physical pixel size (Å)	1.096	1.096	1.096	1.096	1.096	1.096	1.096	1.096	1.096
Symmetry imposed	None (C1)	None (C1)	None (C1)	None (C1)	None (C1)	None (C1)	None (C1)	None (C1)	None (C1)
Initial particle images (no.)	975203	975203	1656248	1656248	2817313	2817313	2817313	2817313	2817313
Final particle images (no.)	53244	53244	637397	955526	218344	133977	169339	271729	230804
Map resolution (Å) FSC threshold = 0.143	3.75	4.07	2.95	2.97	3.39	3.82	3.62	3.45	3.47
Map sharpening B factor (Å²)	117.0	148.3	-80.8	-70.77	129.8	145.2	141.2	119.6	145.9
**Model Refinement**									
Initial model used (PDB ID)	#2–4	1N7S	6MDM	6MDM	#3–4, #13	#3–4, #13	#3–4, #13	#3–4, #13	#3–4, #13
Model composition									
Non-hydrogen atoms	40730	10949	26375	26521	42082	37311	38879	41286	40952
Protein residues	5133	1377	3195	3190	5291	4690	4906	5100	5068
Ligands	13	0	12	11	12	11	12	12	12
B factors (Å²)									
Protein	122.90	105.57	46.17	180.56	287.92	82.07	159.80	76.07	69.85
R.M.S. deviations									
Bond lengths (Å)	0.007	0.0026	0.004	0.004	0.005	0.005	0.005	0.004	0.004
Bond angles (°)	1.220	0.55	0.699	0.799	1.002	1.018	1.066	0.947	0.974
Validation									
MolProbity score	1.40	1.34	1.04	1.31	1.40	1.32	1.31	1.38	1.43
Clashscore	3.58	4.67	2.37	4.69	4.82	4.80	3.61	3.97	5.12
Rotamer outliers (%)	1.03	0	0.76	0.75	0.22	0.75	1.08	0.25	0.30
Ramachandran Statistics									
Favored (%)	96.31	97.50	97.92	97.67	97.20	97.68	97.29	96.76	97.11
Allowed (%)	3.65	2.42	1.98	2.21	2.76	2.28	2.67	3.14	2.77
Outliers (%)	0.04	0.07	0.09	0.13	0.04	0.04	0.04	0.10	0.12

	NSF—αSNAP—2:1 syntaxin:SNAP-25 SNARE complex (substrate bound, non-hydrolyzing; 21bin20S)				NSF—αSNAP—2:2 syntaxin:SNAP-25 SNARE complex (substrate bound, hydrolyzing; 22bin20S)					
	#10 (c23) (EMDB-XXXXX) (PDB YYYY)	#11 (c31) (EMDB-XXXXX) (PDB YYYY)	#12 (c33) (EMDB-XXXXX) (PDB YYYY)	#13 (merge, local) (EMDB-XXXXX) (PDB YYYY)	#14 (c2-11) (EMDB-XXXXX) (PDB YYYY)	#15 (c3-5-6-9) (EMDB-XXXXX) (PDB YYYY)	#16 (c4-7-16-20-24) (EMDB-XXXXX) (PDB YYYY)	#17 (c4-7-16-20-24-m0-sO) (EMDB-XXXXX) (PDB YYYY)	#18 (c4-7-16-20-24-m0-sP) (EMDB-XXXXX) (PDB YYYY)	#19 (c8-14-21) (EMDB-XXXXX) (PDB YYYY)
**Data collection and processing**										
Microscope, camera	Titan Krios, K3	Titan Krios, K3	Titan Krios, K3	Titan Krios, K3	Titan Krios, K3	Titan Krios, K3	Titan Krios, K3	Titan Krios, K3	Titan Krios, K3	Titan Krios, K3
Magnification	22,500×	22,500×	22,500×	22,500×	22,500×	22,500×	22,500×	22,500×	22,500×	22,500×
Voltage (kV)	300	300	300	300	300	300	300	300	300	300
Dataset ID(s)	20190624, 20190919, 20191216	20190624, 20190919, 20191216	20190624, 20190919, 20191216	20190624, 20190919, 20191216	20190816, 20191122	20190816, 20191122	20190816, 20191122	20190816, 20191122	20190816, 20191122	20190816, 20191122
Electron exposure (e–/Å²)	31.560, 36.760, 35.480	31.560, 36.760, 35.480	31.560, 36.760, 35.480	31.560, 36.760, 35.480	33.960, 36.960	33.960, 36.960	33.960, 36.960	33.960, 36.960	33.960, 36.960	33.960, 36.960
Defocus range (μm)	-1.0 to -3.0	-1.0 to -3.0	-1.0 to -3.0	-1.0 to -3.0	-1.0 to -3.0	-1.0 to -3.0	-1.0 to -3.0	-1.0 to -3.0	-1.0 to -3.0	-1.0 to -3.0
Physical pixel size (Å)	1.096	1.096	1.096	1.096	1.096	1.096	1.096	1.096	1.096	1.096
Symmetry imposed	None (C1)	None (C1)	None (C1)	None (C1)	None (C1)	None (C1)	None (C1)	None (C1)	None (C1)	None (C1)
Initial particle images (no.)	2817313	2817313	2817313	2817313	1889717	1889717	1889717	1889717	1889717	1889717
Final particle images (no.)	42789	54516	61150	965222	50776	114008	340359	21214	22586	104003
Map resolution (Å) FSC threshold = 0.143	4.01	3.91	3.69	3.74	4.03	3.65	3.29	3.67	3.52	3.56
Map sharpening B factor (Å²)	114.2	121.7	112.3	221.1	134.8	141.7	136.6	71.4	74.0	134.5
**Model Refinement**										
Initial model used (PDB ID)	#3–4, #13	#3–4, #13	#3–4, #13	1N7S	#3–4, #23	#3–4, #23	#3–4, #23	#3–4, #23	#3–4, #23	#3–4, #23
Model composition										
Non-hydrogen atoms	43495	37457	39769	8953	42375	40071	25898	25042	25413	40590
Protein residues	5466	4673	5027	1130	5376	4992	3205	3165	3206	5126
Ligands	11	11	12	0	13	25	23	17	24	15
B factors (Å²)										
Protein	153.83	93.84	50.83	96.20	155.36	86.04	64.94	116.71	94.71	148.28
R.M.S. deviations										
Bond lengths (Å)	0.004	0.004	0.007	0.004	0.003	0.003	0.003	0.005	0.006	0.004
Bond angles (°)	0.976	0.996	1.116	0.693	0.575	0.619	0.649	1.065	1.147	0.990
Validation										
MolProbity score	1.54	1.69	1.41	1.50	1.77	1.57	1.38	1.68	1.57	1.67
Clashscore	6.78	8.01	4.94	8.23	8.61	5.20	2.90	5.75	4.10	6.98
Rotamer outliers (%)	0.62	1.34	0.58	0.74	0.04	0.47	0.29	0.56	1.10	0.30
Ramachandran Statistics										
Favored (%)	97.05	97.09	97.19	97.75	95.59	95.78	95.67	94.68	95.01	95.93
Allowed (%)	2.82	2.59	2.79	2.16	4.37	4.16	4.27	5.19	4.99	3.95
Outliers (%)	0.13	0.32	0.02	0.09	0.04	0.06	0.06	0.13	0.00	0.12

	NSF—αSNAP—2:2 syntaxin:SNAP-25 SNARE complex (substrate bound, hydrolyzing; 22bin20S)				NSF (substrate free, hydrolyzing)			NSF—αSNAP—minimal 2:2 syntaxin:SNAP-25 SNARE complex (substrate bound, hydrolyzing; min22bin20S)	
	#20 (c13) (EMDB-XXXXX) (PDB YYYY)	#21 (c15) (EMDB-XXXXX) (PDB YYYY)	#22 (c17) (EMDB-XXXXX) (PDB YYYY)	#23 (merge, local) (EMDB-XXXXX) (PDB YYYY)	#24 (c3-24) (EMDB-XXXXX) (PDB YYYY)	#25 (c5-19) (EMDB-XXXXX) (PDB YYYY)	#26 (c6-XXX-22) (EMDB-XXXXX) (PDB YYYY)	#27 (c5-XXX-46) (EMDB-XXXXX) (PDB YYYY)	#28 (c5-XXX-46) (EMDB-XXXXX) (PDB YYYY)
**Data collection and processing**									
Microscope, camera	Titan Krios, K3	Titan Krios, K3	Titan Krios, K3	Titan Krios, K3	Titan Krios, K3	Titan Krios, K3	Titan Krios, K3	Titan Krios, K3	Titan Krios, K3
Magnification	22,500×	22,500×	22,500×	22,500×	22,500×	22,500×	22,500×	22,500×	22,500×
Voltage (kV)	300	300	300	300	300	300	300	300	300
Dataset ID(s)	20190816, 20191122	20190816, 20191122	20190816, 20191122	20190816, 20191122	20190816, 20191122	20190816, 20191122	20190816, 20191122	20200626	20200626
Electron exposure (e–/Å²)	33.960, 36.960	33.960, 36.960	33.960, 36.960	33.960, 36.960	33.960, 36.960	33.960, 36.960	33.960, 36.960	33.992	33.992
Defocus range (μm)	-1.0 to -3.0	-1.0 to -3.0	-1.0 to -3.0	-1.0 to -3.0	-1.0 to -3.0	-1.0 to -3.0	-1.0 to -3.0	-1.0 to -3.0	-1.0 to -3.0
Physical pixel size (Å)	1.096	1.096	1.096	1.096	1.096	1.096	1.096	1.096	1.096
Symmetry imposed	None (C1)	None (C1)	None (C1)	None (C1)	None (C1)	None (C1)	None (C1)	None (C1)	None (C1)
Initial particle images (no.)	1889717	1889717	1889717	1889717	1889717	1889717	1889717	1473284	1473284
Final particle images (no.)	23775	32677	55876	325236	121796	128733	476440	341427	341427
Map resolution (Å) FSC threshold = 0.143	4.50	4.23	3.83	4.14	3.86	3.88	3.15	3.09	3.58
Map sharpening B factor (Å²)	128.9	126.8	119.7	209.8	139.9	141.9	132.3	126.7	164.4
**Model Refinement**									
Initial model used (PDB ID)	#3–4, #23	#3–4, #23	#3–4, #23	1JTH	#3–4	#3–4	#3–4	#3–4, #28	1JTH
Model composition									
Non-hydrogen atoms	42362	42422	25590	11330	29851	26763	29675	40475	13917
Protein residues	5354	5374	3204	1426	3713	3306	3505	4964	1760
Ligands	12	12	17	0	19	20	23	11	0
B factors (Å²)									
Protein	295.12	220.25	91.07	162.68	99.49	91.00	106.79	80.87	70.80
R.M.S. deviations									
Bond lengths (Å)	0.003	0.005	0.004	0.004	0.003	0.004	0.010	0.005	0.004
Bond angles (°)	0.702	1.089	0.799	0.678	0.666	1.062	1.681	1.043	0.556
Validation									
MolProbity score	1.92	1.88	1.75	2.05	1.52	1.69	1.42	1.74	1.56
Clashscore	14.16	9.43	7.33	9.14	4.80	6.39	3.62	4.94	8.66
Rotamer outliers (%)	0.50	1.12	0.73	2.44	0.34	0.28	0.94	2.09	0.54
Ramachandran Statistics									
Favored (%)	96.11	94.97	95.07	96.03	96.00	95.14	96.08	96.44	97.57
Allowed (%)	3.70	4.90	4.74	3.62	3.91	4.58	3.75	3.31	2.31
Outliers (%)	0.19	0.13	0.19	0.35	0.09	0.28	0.17	0.24	0.12

Supplemental Table 1 | Cryo-EM data, map, and model statistics

Data, map, and model statistics for the 25 NSF, sx20S, 21bin20S, 22bin20S, and α-SNAP—SNARE subcomplex models.

NSF D1 Model Features

Index	Model	Buffer Nucleotide	EDTA or Mg	Spire SNARE complex	SNARE in D1 pore	F engaged	A	B	C	D	E	F
1	20s_sx1a-tet_atp-edta_c20-33_full_model.pdb	ATP	EDTA	syntaxin H3 SNARE tetramer	syntaxin-1a	✓	ADP	ATP	ATP	ATP	ATP	ATP
3	20s_binary_atp-edta_c4-3-5-3_full_model.pdb	ATP	EDTA	?	?	✓	ADP	ATP	ATP	ATP	ATP	ATP
4	20s_binary_atp-edta_c4-3-5-4_full_model.pdb	ATP	EDTA	?	?	☐	ADP	ATP	ATP	ATP	ATP	Apo
5	20s_binary_atp-edta_c4-27-30-46_full_model.pdb	ATP	EDTA	Full 2:1 binary complex (21bin20S)	syntaxin-1a	✓	ADP	ATP	ATP	ATP	ATP	ATP
6	20s_binary_atp-edta_c5-47_full_model.pdb	ATP	EDTA	Full 2:1 binary complex (21bin20S)	syntaxin-1a	☐	ADP	ATP	ATP	ATP	ATP	Apo
7	20s_binary_atp-edta_c7-25-34-45_full_model.pdb	ATP	EDTA	Full 2:1 binary complex (21bin20S)	syntaxin-1a	✓	ADP	ATP	ATP	ATP	ATP	ATP
8	20s_binary_atp-edta_c14-15-20-42_full_model.pdb	ATP	EDTA	Full 2:1 binary complex (21bin20S)	syntaxin-1a	✓	ADP	ATP	ATP	ATP	ATP	ATP
9	20s_binary_atp-edta_c22-35-37-39_full_model.pdb	ATP	EDTA	Full 2:1 binary complex (21bin20S)	SNAP-25	✓	ADP	ATP	ATP	ATP	ATP	ATP
10	20s_binary_atp-edta_c23_full_model.pdb	ATP	EDTA	Full 2:1 binary complex (21bin20S)	syntaxin-1a	☐	ADP	ATP	ATP	ATP	ATP	Apo
11	20s_binary_atp-edta_c31_full_model.pdb	ATP	EDTA	Full 2:1 binary complex (21bin20S)	SNAP-25	☐	ADP	ATP	ATP	ATP	ATP	Apo
12	20s_binary_atp-edta_c33_full_model.pdb	ATP	EDTA	Full 2:1 binary complex (21bin20S)	syntaxin-1a	✓	ADP	ATP	ATP	ATP	ATP	ATP
14	20s_binary_atp-mg_c2-11_full_model.pdb	ATP	Mg	Full 2:2 binary complex (22bin20S)	syntaxin-1a	☐	ADP	ADP	ADP	ADP + Pi	ATP	ADP
15	20s_binary_atp-mg_c3-5-6-9_full_model.pdb	ATP	Mg	Full 2:2 binary complex (22bin20S)	syntaxin-1a	☐	ADP	ADP + Mg	ADP + Pi + Mg	ADP + Pi + Mg	ATP + Mg	ADP
16	20s_binary_atp-mg_c4-7-16-20-24_full_model.pdb	ATP	Mg	?	?	☐	ADP	ADP	ADP + Pi + Mg	ADP + Pi + Mg	ATP + Mg	ADP
17	20s_binary_atp-mg_c4-7-16-20-24-m0-sO_full_model.pdb	ATP	Mg	?	?	✓	ADP	ADP	ADP + Mg	ADP + Pi + Mg	ATP + Mg	ATP
18	20s_binary_atp-mg_c4-7-16-20-24-m0-sP_full_model.pdb	ATP	Mg	?	?	☐	ADP	ADP + Mg	ADP + Pi + Mg	ADP + Pi + Mg	ATP + Mg	ADP
19	20s_binary_atp-mg_c8-14-21_full_model.pdb	ATP	Mg	Full 2:2 binary complex (22bin20S)	syntaxin-1a	☐	ADP	ADP + Pi + Mg	ADP + Pi + Mg	ADP + Pi + Mg	ATP + Mg	ADP
20	20s_binary_atp-mg_c13_full_model.pdb	ATP	Mg	Full 2:2 binary complex (22bin20S)	syntaxin-1a	☐	ADP	ADP	ADP	ADP	ATP	ADP
21	20s_binary_atp-mg_c15_full_model.pdb	ATP	Mg	?	?	☐	ADP	ADP	ADP	ADP	ATP	ADP
22	20s_binary_atp-mg_c17_full_model.pdb	ATP	Mg	Full 2:2 binary complex (22bin20S)	?	☐	ADP	ADP	ADP + Pi	ADP + Pi	ATP + Mg	ADP
23	20s_binary_atp-mg_c19_full_model.pdb	ATP	Mg	?	?	☐	ADP	ADP + Pi	ADP + Pi	ADP + Mg	ATP	ADP
24	apo_binary_atp-mg_c3-24_full_model.pdb	ATP	Mg	None	None	N/A	ADP	ADP	ADP + Pi	ADP + Pi	ADP + Pi	ADP
25	apo_binary_atp-mg_c5-19_full_model.pdb	ATP	Mg	None	None	N/A	ADP	ADP	ADP + Pi	ADP + Pi	ADP	ADP
26	apo_binary_atp-mg_c6-11-13-17-18-22_full_model.pdb	ATP	Mg	None	None	N/A	ADP	ADP + Pi	ADP + Pi	ADP + Pi	ADP + Pi	ADP
27	20s_binary-mini_atp-edta_c5-XXX-46_full_model.pdb	ATP	EDTA	Minimal 2:2 binary complex (min22bin20S)	SNAP-25	☐	ADP	ATP	ATP	ATP	ATP	Apo

Supplemental Table 2 | Cryo-EM class reference

Model details related to substrate engagement and nucleotide state for the 25 NSF, sx20S, 21bin20S, and 22bin20S models.

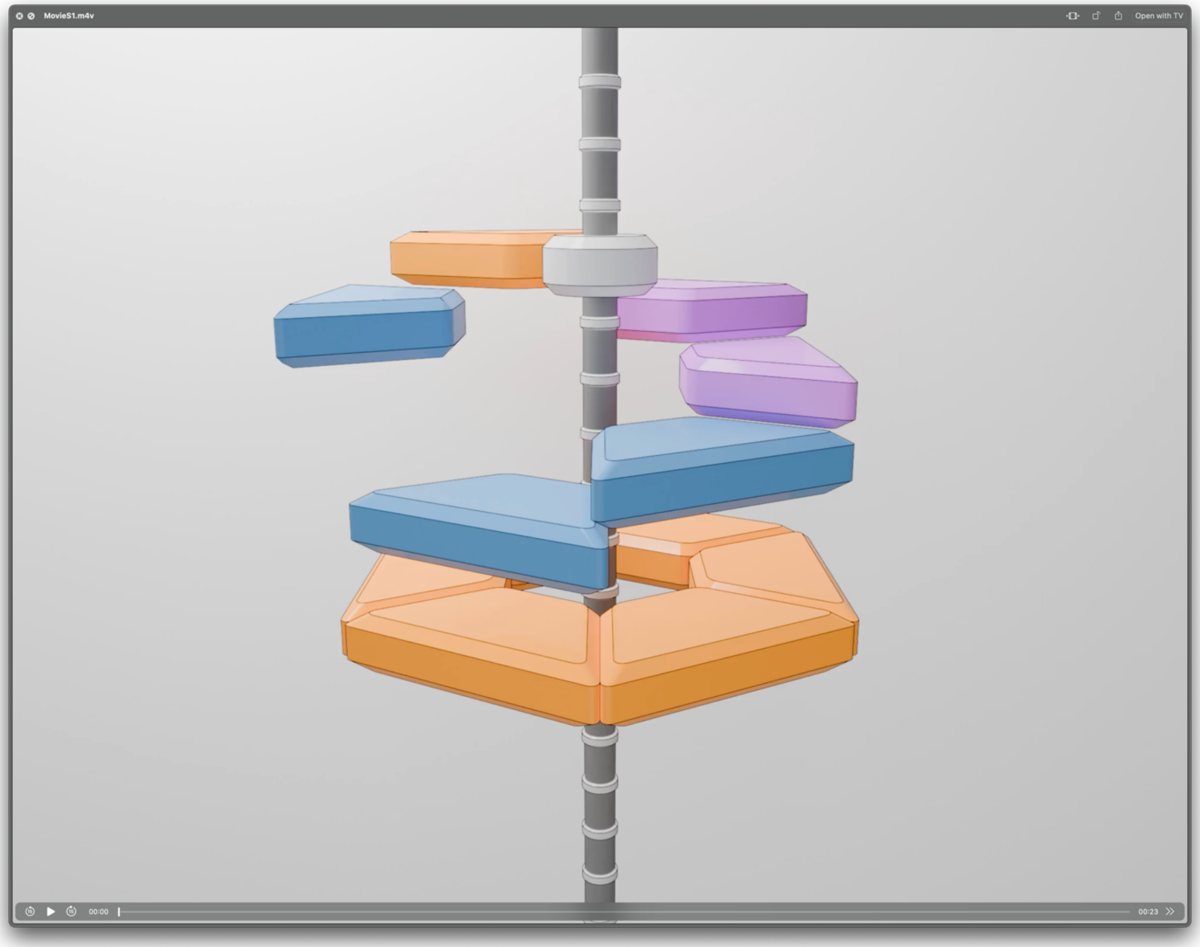

Supplemental Movie 1| A model of NSF motions and substrate translocation associated with nucleotide rebinding and hydrolysis. A sequential hydrolysis model for SNARE complex disassembly by NSF inferred from comparison of different classes from non-hydrolyzing and hydrolyzing conditions. Following hydrolysis-independent substrate side loading, the D1 ring is found in a pre-hydrolysis state with ATP in protomer E of D1. Protomer F nucleotide exchange then enables ATP binding, re-engagement to both SNARE substrate, and the formation of the interprotomer interface with protomer E, triggering ATP hydrolysis in protomer E and substrate translocation. This is accompanied by P_i and Mg^{2+} release from down-ring protomers; in many structures of the different classes, this apparent release occurs between protomers C and B. The cycle then begins anew. Blue corresponds to ADP-bound, red to ATP-bound, purple to ADP•P_i bound, and grey to nucleotide-free. See Fig. 7B for key frames and further detail.
